# ERCC6L facilitates the onset of mammary neoplasia and promotes the high malignance of breast cancer by accelerating the cell cycle

**DOI:** 10.1186/s13046-023-02806-x

**Published:** 2023-09-04

**Authors:** Hong Yang, Xiangjin Zhen, Yihui Yang, Yizhi Zhang, Sen Zhang, Yue Hao, Guanhua Du, Hongquan Wang, Bailin Zhang, Wan Li, Jinhua Wang

**Affiliations:** 1 The State Key Laboratory of Bioactive Substance and Function of Natural Medicines, Beijing, 100050 China; 2 Key Laboratory of Drug Target Research and Drug Screen, Institute of Materia Medica, Chinese Academy of Medical Science and Peking Union Medical College, Beijing, 100050 China; 3 Department of Pancreatic Cancer, Tianjin’s Clinical Research Center for Cancer, Key Laboratory of Cancer Prevention and Therapy, Tianjin Medical University Cancer Institute and Hospital, National Clinical Research Center for Cancer, Tianjin, 300060 China; 4 Cancer Hospital, Chinese Academy of Medical Sciences, Beijing, 100021 China

**Keywords:** Breast cancer, ERCC6L, Cell mitosis, Conditional knockout mice, KIF4A

## Abstract

**Background:**

Breast cancer (BC) is the leading cause of morbidity and the second leading cause of death among female malignant tumors. Although available drugs have been approved for the corresponding breast cancer subtypes (ER-positive, HER2^+^) currently, there are still no effective targeted drugs or treatment strategies for metastatic breast cancer or triple-negative breast cancer that lack targets. Therefore, it is urgent to discover new potential targets. ERCC6L is an essential protein involved in chromosome separation during cell mitosis. However, the effect of ERCC6L on the tumorigenesis and progression of breast cancer is unclear.

**Methods and results:**

Here, we found that ERCC6L was highly expressed in breast cancer, especially in TNBC, which was closely related to poor outcomes of patients. An ERCC6L conditional knockout mouse model was first established in this study, and the results confirmed that ERCC6L was required for the development of the mammary gland and the tumorigenesis and progression of mammary gland cancers. In in vitro cell culture, ERCC6L acted as a tumor promoter in the malignant progression of breast cancer cells. Overexpression of ERCC6L promoted cell proliferation, migration and invasion, while knockdown of ERCC6L caused the opposite results. Mechanistically, ERCC6L accelerated the cell cycle by regulating the G_2_/M checkpoint signalling pathway. Additionally, we demonstrated that there is an interaction between ERCC6L and KIF4A, both of which are closely related factors in mitosis and are involved in the malignant progression of breast cancer.

**Conclusions:**

We first demonstrated that ERCC6L deficiency can significantly inhibit the occurrence and development of mammary gland tumors. ERCC6L was found to accelerate the cell cycle by regulating the p53/p21/CDK1/Cyclin B and PLK/CDC25C/CDK1/Cyclin B signalling pathways, thereby promoting the malignant progression of breast cancer cell lines. There was a direct interaction between KIF4A and ERCC6L, and both are closely associated with mitosis and contribute to growth and metastasis of breast tumor. To sum up, our results suggest that ERCC6L may be used as a promising target for the treatment of BC.

**Supplementary Information:**

The online version contains supplementary material available at 10.1186/s13046-023-02806-x.

## Introduction

Breast cancer (BC) is a malignant tumor occurring in the glandular epithelium of the breast. According to the latest cancer statistics released by CA (A Cancer Journal for Clinicians), the incidence rate of breast cancer ranks first among female malignant tumors (297,790/948,000) and has been on the rise since the mid-2000s (an annual increase of approximately 0.5%). In addition, breast cancer is the second leading cause of cancer death in women (43,170/287,740) [1]. BC can be divided into luminal A, luminal B, HER2-enriched, basal-like and triple-negative (ER^−^, PR^−^, HER2^−^) subtypes based on the gene expression profile [2]. Although there are marketed drugs for some BC subtypes, metastatic BC is still considered incurable [3]. Triple-negative breast cancer (TNBC), which is highly aggressive and malignant and has the worst prognosis, has no specific targeted drugs due to the lack of therapeutic targets. Therefore, it is still necessary to discover novel potential targets for breast cancer, especially those lacking therapeutic targets, such as TNBC.

Cell division is the process of dividing cells into two, which is of great significance for growth, tissue repair and regeneration, and asexual reproduction of single cells. It is a strictly regulated and highly ordered process that includes interphase and M phases, monitored by inherent cycle checkpoints within the cell to ensure the fidelity of cell replication [4]. It was reported that most breast cancers (especially in later stages of the disease) exhibited some degree of abnormal genomic structure, such as aneuploidy chromosomes, which may be related to genetic defects of mitotic spindle examination sites [5]. Therefore, targeting those defects to further induce tumor cell death may be a potentially desirable strategy. In addition, a variety of poly ADP-ribose polymerase (PARP) inhibitors have been approved for the treatment of metastatic breast cancer [6, 7]. Inhibiting PARP can cause impaired DNA single strand break repair and damage the stability of replication forks, thereby inducing DNA damage. During mitosis, the accumulation of damaged DNA further leads to abnormal chromosome segregation, cytokinesis failure, polykaryocytosis, and ultimately cell death, further suggesting the potential to target the unique cell cycle progression of BC to interfere with tumorigenesis and progression.

ERCC Exception Repair 6 Like (ERCC6L), belonging to the SWItch/Sucrose Non-Fermentable (SWI/SNF2) protein family, which is also named PLK1-interacting checkpoint helicase (PICH), is a DNA helicase closely related to cell mitosis [8]. It is an essential component of the spindle assembly checkpoint (one of the important mechanisms to ensure proper chromosome separation during mitosis). Moreover, ERCC6L, as a DNA transposase, can translocate to the endocentric and kinetochore regions of mitotic chromosomes to regulate spindle assembly checkpoint signals [9] and can also bind to ribosome DNA and ultrafine DNA bridges (UFBs) to participate in sister chromatid separation during mitosis. In addition, ERCC6L carries an SNF2-like ATPase domain, and its ATPase activity is required for the decomposition of UFBs before cytokinesis [10, 11]. Therefore, ERCC6L plays an important role in cell survival and proliferation.

Some studies reported that the deletion of ERCC6L could induce chromosomal abnormalities (chromatin bridges, micronuclei, dikaryon, etc.), embryonic lethality, DNA damage, p53 activation, and increased apoptosis [12, 13]. These results, to some extent, revealed the feasibility of targeting ERCC6L. ERCC6L was also reported to be abnormally expressed in various tumors, such as renal cell carcinoma, liver cancer, and laryngeal squamous cell carcinoma, and was involved in processes, such as cell proliferation, migration, and invasion [14–16]. Huang et al. [17] reported the high expression of ERCC6L and its potential role in TNBC. However, there is still little information about the application of ERCC6L knockout mouse models in BC. Moreover, the role of ERCC6L in BC tumorigenesis and progression remains unclear. Therefore, further exploration is warranted.

In this study, we first demonstrated the potential function of ERCC6L using an ERCC6L conditional knockout mouse model. Compared with wild-type mice, ERCC6L deletion delayed mammogenesis. By hybridizing ERCC6L conditional knockout mice with MMTV-PyMT mice (a mouse model in which specific expression of polyomavirus middle T antigen (PyMT) driven by the mouse mammary virus MMTV could rapidly facilitate multifocal mammary tumor formation), it was found that ERCC6L deficiency can significantly inhibit the occurrence and development of mammary gland tumors. Mechanistically, ERCC6L could accelerate the cell cycle by regulating the p53/p21/CDK1/Cyclin B and PLK/CDC25C/CDK1/Cyclin B signalling pathways, thereby promoting the malignant progression of breast cancer cell lines. Additionally, we also revealed the potential relationship between KIF4A and ERCC6L, that is, there was a direct interaction between KIF4A and ERCC6L, and both are closely associated with mitosis and contribute to tumor growth and metastasis. Taken together, our results suggest that ERCC6L plays a vital role in the occurrence and development of BC and might be a potential target for BC treatment.

## Materials and methods

### Cell culture and tumor specimens

The BC cell lines ZR-75-1, MCF-7, T47D, BT474, HCC1937, MDA-MB-231, MDA-MB-435, MDA-MB-453, MDA-MB-468, and SKBR3 were purchased from American Type Culture Collection (ATCC). Dulbecco’s modified Eagle’s medium (DMEM) and RPMI 1640 (Gibco, CA, USA) with 10% foetal bovine serum (FBS) were used to culture MCF-7, MDA-MB-231, MDA-MB-435, MDA-MB-453, MDA-MB-468, SKBR3 and BT474, HCC1937, T47D, ZR-75-1 cells. The serum was purchased from Procell Life Science & Technology (Wuhan, China). All cells were cultured in a humidified incubator with 5% CO_2_ at 37 °C. The human BC and adjacent normal tissues used in this article were gifted by Dr. Bailin Zhang (National Cancer Center/National Clinical Research Center for Cancer/Cancer Hospital, Chinese Academy of Medical Sciences and Peking Union Medical College) and were approved by the Institutional Review Board (IRB).

### Construction of stable cell lines and transfection

For the construction of MCF-7 and T47D cell lines stably overexpressing ERCC6L, control and ERCC6L lentiviruses were used to infect both cell lines for 72 h, followed by selection with 3 µg/mL puromycin. For stable silencing of ERCC6L, MDA-MB-231 cells were infected with control, shERCC6L#1 and shERCC6L#2 lentiviruses. Subsequently, they were screened with 3 µg/mL puromycin. For transient overexpression of KIF4A, the human full-length KIF4A cDNA clone was transfected into 231 cell lines using Lipofectamine 3000 reagent in accordance with the manufacturer’s instructions.

### Transgenic mouse model and tumor monitoring

The ERCC6L^flox/flox^ and MMTV-Cre/Line D mice provided by Biocytogen Pharmaceuticals Co., Ltd. Mice were kept in the Laboratory Animal Center, Institute of Materia Medica, Chinese Academy of Medical Sciences under 12 h/d light, 22 ± 3 °C temperature and 50 ± 5% relative humidity. All experimental conditions and experiments performed on animals were in accordance with the guidelines of the Animal Ethics Committee of the Institute of Materia Medica, Chinese Academy of Medical Sciences (Ethics number: 00008101).

The flox-Cre recombination system was applied to generate mice with mammary epithelial cell-specific ERCC6L deletion. ERCC6L^flox/wt^ Cre^+/−^ mice were generated by crossing ERCC6L^flox/flox^ mice with MMTV-Cre mice. Then, ERCC6L^flox/wt^ Cre^+/−^ mice were hybridized with ERCC6L^flox/y^ Cre^+/−^ mice and ERCC6L^wt/y^ Cre^+/−^ mice to obtain ERCC6L^flox/flox^ Cre^+/−^ mice and ERCC6L^wt/wt^ Cre^+/−^ mice, respectively. At 6, 8 and 12 weeks, the fourth groin mammary glands of female mice were weighed, and a whole mount assay was performed.

To generate PyMT mice, we crossbred female ERCC6L mice of different genotypes with MMTV-PyMT mice. At 8 weeks, whole mount staining was performed on the fourth groin mammary gland of female mice to evaluate multifocal dysplasia. To assess palpable tumor onset, animals were palpated weekly. After the formation of palpable tumors, the body weight of mice was recorded, and tumor volume was measured using Vernier callipers within 42 days. At 16 weeks, the mice were dissected, and their body weight, the number of tumors per mouse, and the wet weight of each tumor were recorded. Tumor tissues, lungs, and spleens were soaked in 4% paraformaldehyde (PFC) for immunohistochemistry (IHC) and immunofluorescence (IF). The genotypes of all female mice were identified by specific PCR analysis of mouse tail genomic DNA. Primer information is listed in Supplementary Table 1.

### Nude mouse axillary xenograft tumor model

Female BALB/c-nu nude mice, purchased from Beijing Vital River Laboratory Animal Technology, were divided into three groups (n = 6) and separately injected with MDA-MB-231 shNC, MDA-MB-231 shERCC6L#1, and MDA-MB-231 shERCC6L#2 cells (1 × 10^7^/0.1 mL). After the tumor had grown (less than 1000 mm^3^), each mouse was dissected to measure the volume of the tumor. The calculation formula is V = 0.5xLxW^2^ mm^3^ (L represents length and W represents width, Unit: mm).

### Whole mount staining

The fourth inguinal mammary gland was removed, spread on a glass slide, and air-dried for 5–10 min. Fixation was performed with Carnoy’s solution (60% ethanol, 30% chloroform, and 10% glacial acetic acid) for 2–4 h. The slides were then immersed in gradient concentrations of ethanol (75%-50%-30%) for 15 min. After immersion in ddH_2_O for 5 min, the slides were immersed in a container with dye liquor (0.2% carmine and 0.5% aluminium potassium sulfate) for 1–2 days. They were then decolorized with 70% ethanol containing 2% hydrochloric acid for 2 h, immersed in gradient concentrations of ethanol (70-95%-100%) for 15 min, defatted with xylene for 20 min, and finally stored in methyl salicylate.

### Cell viability, migration and invasion assays

Cell Counting Kit-8 (CCK-8) assays were carried out to check cell proliferation. The cells were first seeded in 96-well plates and then incubated in incubators for 0, 24, 48, 72 and 96 h. Finally, the plates were placed in SpectraMax M5, and the absorbance value of each well was measured at 450 nm for quantification. Relative cell proliferation rate (100%) = (OD_24/48/72 h/96 h_-OD_0h_)/OD_0h_. In terms of migration and invasion assays, the cells were counted and inoculated in 8 μm polycarbonate membranes in Costar 24-Transwell plates. Then, after being cultured for 3–4 h (after cell attachment), serum-free medium and 10% FBS medium were added to the upper and bottom chambers, respectively. After 19 h (for migration) and 22 h (for invasion), cells were fixed with 4% paraformaldehyde (PFA) and stained with 1% crystal violet. Subsequently, after cells in the upper chamber were wiped off, the chambers were placed under a microscope for observation. Note that in the invasion assay, the upper chambers were coated with Matrigel™ diluted in serum-free medium (1:7) before seeding the cells.

### Soft agar colony formation assay and 3D Matrigel cell culture

The low melting point agarose was prepared at 3.5% mother liquor, which needed to be autoclaved before use. The stock solution was diluted 1:5 (0.7% agar) and 1:10 (0.35% agar) and successively spread in 6-well plates to form the bottom and upper layers. The upper layer contained prepared cells (approximately 3000/well). Before being placed into the incubator, plates were first placed in a 4 °C refrigerator for 30 min. After being plated in an incubator for 2–3 weeks, cells were stained with MTT solution (5 mg/mL) and then counted. For the 3D Matrigel assay, the cell suspension and Matrigel were mixed in a 1:1 ratio and inoculated in 96-well plates. After two weeks, clones were observed and photographed.

### Cell proliferation EdU assay

Cells were plated in 96-well plates. EdU reagent (5-Ethynyl-2’-deoxyuridine) (1:1000 dilution) was added to plates and incubated for 2 h. The cells were then fixed with 4% PFA for 30 min, washed with 2 mg/mL glycine solution and PBS 2–3 times, and then permeabilized with PBS with 0.5% Triton X. After permeabilization, the prepared 1x Apollo staining solution and Hoechst 33,342 staining solution were added successively, and the cells were incubated in the dark at room temperature for 30 min. Finally, the cells were observed under a microscope.

### Flow cytometry (FCM) for cell cycle assay

The cells were seeded in 60-mm medium dishes. After 48 h, cells were collected, washed with PBS, and fixed overnight with 70% ethanol at 4 °C. Then, the cells were washed again with PBS and incubated successively in PBS with RNase (20 µg/mL) and PBS with 50 µg/mL PI and 0.5% Triton X for 30 min at room temperature in the dark. Finally, the cell suspension was filtered through a 300-mesh screen, and its fluorescence was detected using Accuri C6. FlowJo v10 was used for data processing.

### Immunohistochemistry (IHC) and immunofluorescence (IF)

Human BC tissue samples and animal tissues were prefixed and made into paraffin sections. After roasting, dewaxing, and hydration, the sections were infiltrated with preheated blocking permeabilizing solution (PBS with 30% H_2_O_2_ and Triton X) for 30 min to reduce endogenous peroxidase activity. Subsequently, the sections were subjected to antigen repair and blocked using sodium citrate buffer (pH 6.0) and serum. ERCC6L, KIF4A, Ki67, F4/80, and CD31 (1:200 dilution) were added to the tissue sections for incubation at 4 °C overnight. The next day, sections were washed and incubated with secondary antibodies for 1–2 h at 37 °C. Diaminobenzidine (DAB), Mayer’s Haematoxylin Stain Solution and neutral balata were subsequently applied to stain, counterstain (negative controls were treated with rabbit serum) and seal slides. Finally, the stained sections were photographed under a Leica TCS SP8X confocal microscope. The staining density and positive rate of protein were analyzed by Image J (V1.8.0).

The cells were preseeded onto glass bottom cell culture dishes, and 24 h later, the cells were fixed with 4% PFA for 20 min, permeabilized with 0.3% Triton X for 10 min, and blocked with 5% BSA for 30 min at room temperature. At the end of blocking, diluted KIF4A (1:100) and ERCC6L (1:100) antibodies were added to the bottom of the dish, and the cells were incubated overnight at 4 ℃. Subsequently, the cells were stained with the corresponding fluorescent secondary antibodies for 1 h at room temperature (Alexa 488 for ERCC6L and Alexa 574 for KIF4A). Finally, the cells were further stained with DAPI for 30 min. A confocal microscope was used to acquire images.

### Protein extraction and immunoblotting analysis by Western blotting (WB)

Total proteins in human BC tissue samples, animal tissues and cells were extracted by RIPA lysis buffer with protease and phosphatase inhibitors (CwBio, Beijing) and subsequently quantified by the BCA method. For immunoblotting, equal amounts of proteins were electrophoresed on 8-10% SDS‒PAGE gels and subsequently transferred to PVDF membranes. After blocked the membranes with 5% BSA or 5% defatted milk for 1 h at room temperature, the membranes were incubated with primary antibody overnight at 4 °C. After being washed with TBST, membranes were incubated with commensurable secondary antibodies. Finally, the membrane was cleaned again with TBST and then exposed in Tanon 5200 (Shanghai, China) by dropping ECL regents on it. Data were analyzed by Image J V1.8.0. The antibodies used are shown in Supplementary Table 1.

### Coimmunoprecipitation (Co-IP)

Cells seeded into 100 mm dishes were lysed using IP lysis buffer and centrifuged to obtain the supernatant. Part of the supernatant could be directly applied for Western blotting after denaturation. For Co-IP, the supernatant was mixed with protein A/G agarose beads and anti-DDK beads and then rotated overnight at 4 °C. Subsequently, the beads were washed, mixed with 2x SDS‒PAGE loading buffer, and heated at 95 °C for 5 min for Western blotting analysis.

### Statistical analysis

The mean ± standard deviation (SD) was used to present the results. *P* values were used to evaluate the significance of the differences, which were calculated by unpaired two-tailed Student’s t test or one-/two-way ANOVA. There were statistically significant differences when *P* values < 0.05.

## Results

### ERCC6L is highly expressed in BC patients and associated with poor outcome

**Fig. 1:**
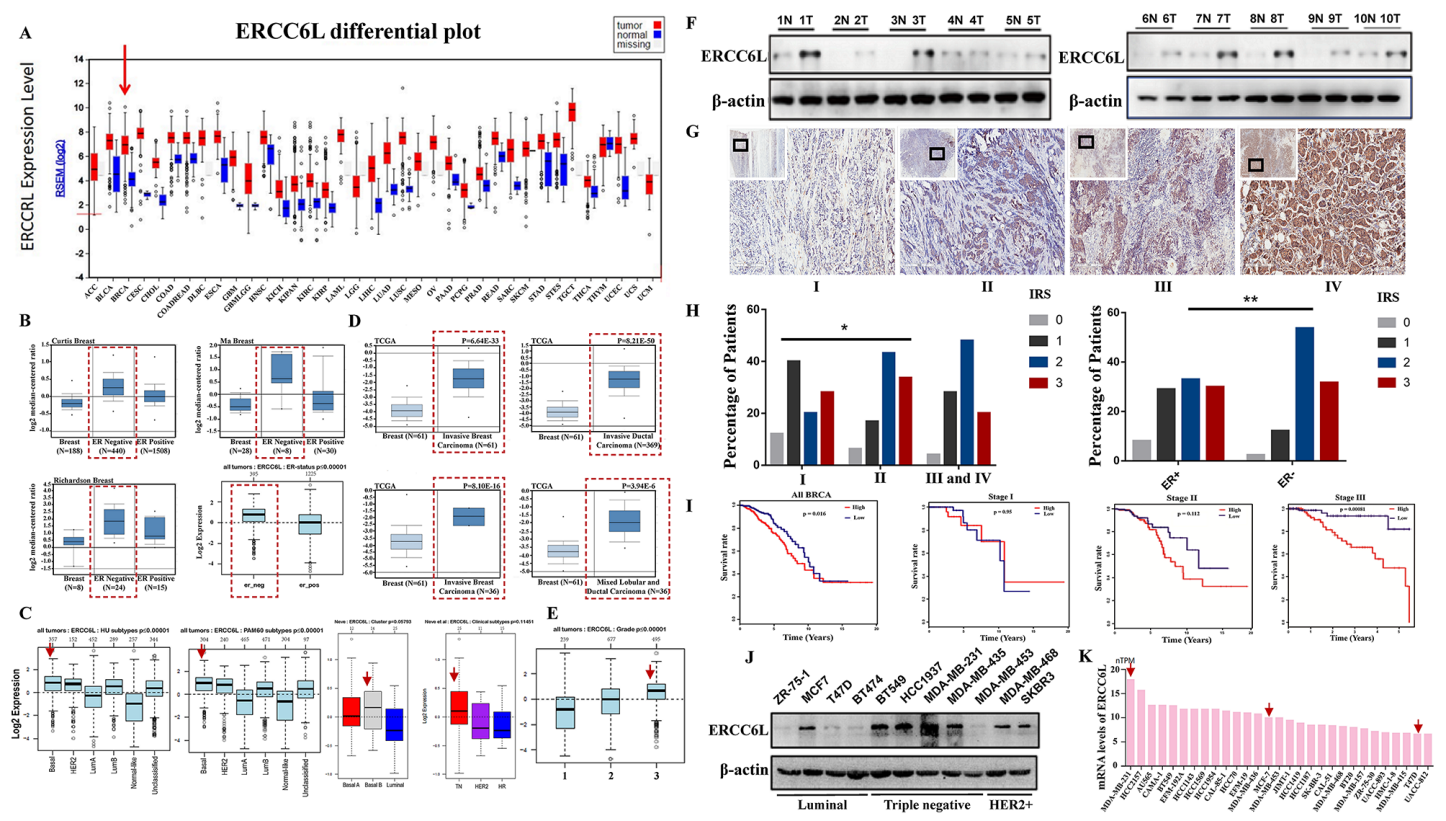
ERCC6L is highly expressed in BC patients and associated with poor outcome. (**A**) The expression profile of ERCC6L in different tumors was analyzed using a website (https://ualcan.path.uab.edu/), and the ERCC6L level was higher in BC than in normal tissues. (**B**) ERCC6L expression was upregulated in ER-negative breast cancer tissues using datasets from the Oncomine website (www.oncomine.org). Squares represent ER^−^ breast cancer patients. (**C**) The mRNA level of ERCC6L was increased in basal-like breast cancer and TNBC based on the Oncomine database. Arrows represent patients with basal-like or triple-negative breast cancer. (**D**) The Oncomine database showed that the mRNA level of ERCC6L was increased in invasive breast carcinoma. Squares represent patients with more aggressive breast cancer. (**E**) ERCC6L expression was positively correlated with the clinical stage of BC. Arrow represents patients with grade III breast cancer. (**F**) The expression level of ERCC6L protein was higher in breast tumor tissue samples (T) than in adjacent non-tumor tissues (N) (n = 10). (**G**) ERCC6L protein levels were positively correlated with clinical stages by analyzing samples from 145 breast cancer patients. (**H**) ERCC6L expression in different stages of breast cancer patients and in ER^+^/ER^−^ patients. IRS: immunoreactive scores. Student’s t test or one-way ANOVA was applied to analyse the statistical significance. ***P < 0.001, **P < 0.01, *P < 0.05. (**I**) Survival curves of patients with different grades of BC. (**J**) ERCC6L protein levels in eleven breast cancer cell lines. (**K**) mRNA levels of ERCC6L in different BC cell lines. Data from the HPA website (https://www.proteinatlas.org/). Arrows refer to the three cell lines used in the subsequent experiments. The results from Western blotting are representative of three independent experiments, and the signals were quantified by densitometry

To evaluate the expression of ERCC6L in BC and whether it is associated with the progression of BC, *in silico* assays were performed using the UALCAN (https://ualcan.path.uab.edu/), Oncomine (www.oncomine.org), and GEPIA (http://gepia.cancer-pku.cn/) databases. The results showed that ERCC6L was highly expressed in BC regardless of the BC subtype compared with the normal controls (Fig. 1A and Supplementary Fig. S1a). Among them, ER-negative patients had higher ERCC6L expression levels than ER-positive patients (Fig. 1B). The mRNA levels of ERCC6L in patients with basal-like breast cancer or triple-negative breast cancer were higher than those in patients with other subtypes of breast cancer (Fig. 1C and Supplementary Fig. S1b-c). Additionally, as the grade of BRAC increased, ERCC6L presented a higher expression level, which was closely related to the aggressiveness and poor prognosis of BC patients (Fig. 1D-E and Supplementary Fig. S1d-f). Therefore, we speculated that ERCC6L might be involved in the malignant progression of breast tumors. Subsequently, we determined the level of ERCC6L in human BC tissue samples and its prognostic value in BC patients. Ten pairs of tumor tissue samples and adjacent normal breast tissues from BC patients were used to assess the expression of ERCC6L by Western blotting, and the results showed that ERCC6L was more highly expressed in BC tissues than in normal controls (Fig. 1F and Supplementary Fig. S1g). Moreover, tumor specimens from 145 breast cancer patients were also examined by immunohistochemistry (IHC), and representative pictures are shown in Fig. 1G and Supplementary Fig. S1h, which indicated that the expression of ERCC6L gradually increased with increasing BC grade. In addition, among patients with an IRS score of 2, a higher proportion of patients with high ERCC6L expression were found in ER-negative BC patients (53.7%) than in ER-positive BC patients (33%) (Fig. 1H). The results from IHC also showed that the expression level of ERCC6L was positively correlated with the grade of BC and a worse prognosis in BC patients (Fig. 1I). All these results were consistent with the results of *in silico* assays, as mentioned above. We also checked the expression of ERCC6L in 11 BC cell lines (6 non-triple-negative and 5 triple-negative cell lines) and found that most BC cell lines had high expression levels of ERCC6L. It is worth mentioning that triple-negative BC cell lines had an overall higher expression level of ERCC6L (Fig. 1J). This result was consistent with ERCC6L mRNA data from the Human Protein Atlas (HPA) database (https://www.proteinatlas.org/) (Fig. 1K). Taken together, these results showed that ERCC6L was highly expressed in BC patients and closely associated with a malignant degree of BC and poor prognosis for BC patients.

### ERCC6L promoted the development of breast cancer in vivo and in vitro

**Fig. 2:**
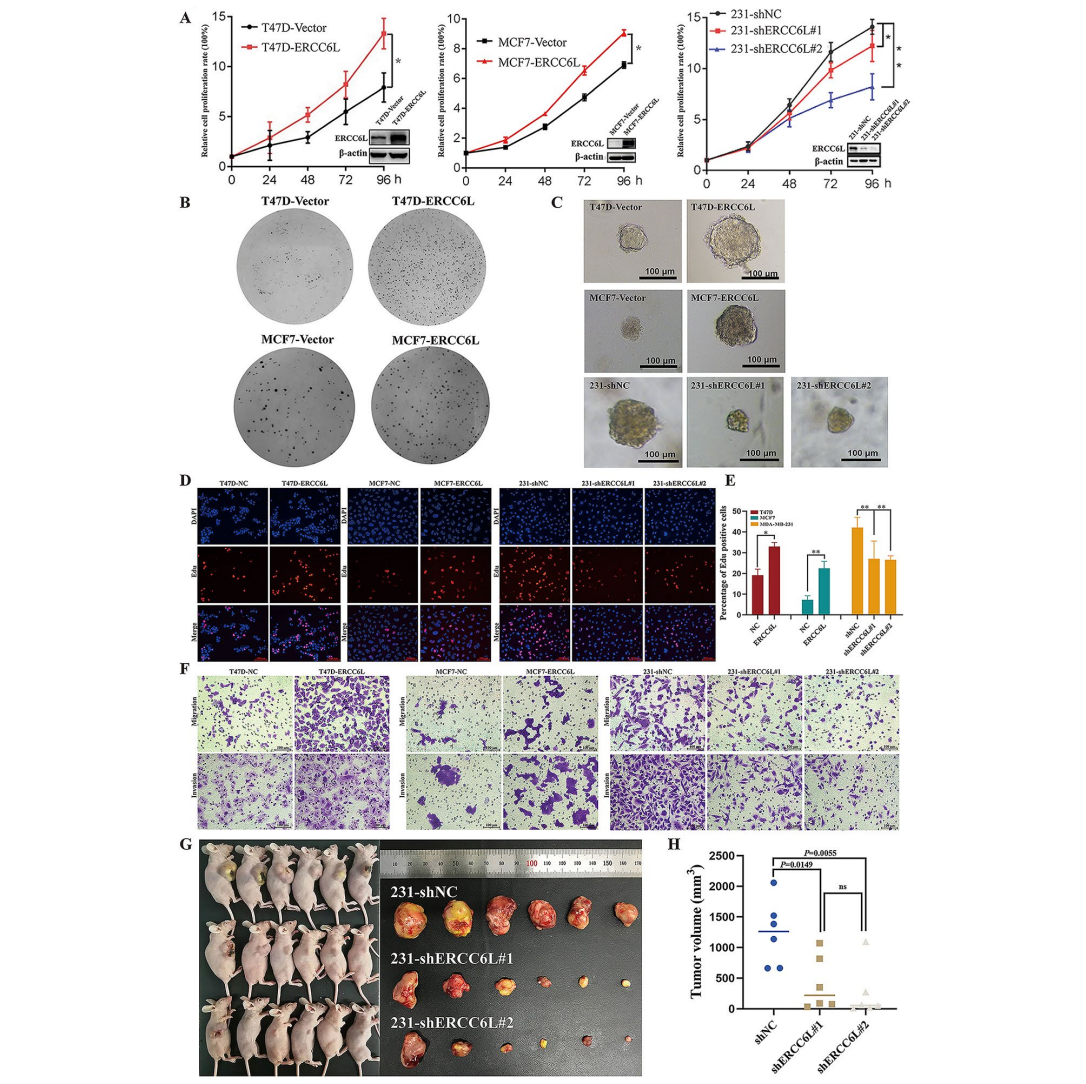
ERCC6L promoted the development of breast cancer in vivo and in vitro. (**A**) Relative growth of T47D, MCF-7, and MDA-MB-231 cells at 24, 48, 72 and 96 h. (**B**) Overexpression of ERCC6L promoted colony formation of T47D and MCF-7 cells. (**C**) Cell growth in 3D Matrigel of T47D, MCF-7, and MDA-MB-231 cells. The scale bar is 100 μm. (**D**) Overexpression of ERCC6L facilitated DNA synthesis in T47D and MCF-7 cells, while ERCC6L knockdown suppressed DNA synthesis in MDA-MB-231 cells. The scale bar is 100 μm. (**E**) Quantitative analysis of the EdU assay. (**F**) Overexpression of ERCC6L promoted the migration and invasion of T47D and MCF-7 cells, whereas ERCC6L knockdown had the opposite effects. (**G**) Images of xenograft nude mice and loaded tumors in different groups. The scale bar is 100 μm. (**H**) ERCC6L knockdown significantly reduced the tumor volume. All experiments were repeated three times and then quantified. Means ± SDs were used to represent data. Student’s t test or one-way ANOVA was applied to analyze the statistical significance. ***P < 0.001, **P < 0.01, *P < 0.05

To explore the functional role of ERCC6L in BC, loss- and gain-of-function assays were carried out. The expression of ERCC6L in MDA-MB-231 cells was silenced using two distinct shRNA lentiviral targeting constructs (named 231 shERCC6L#1 and 231 shERCC6L#2). Control cells infected with scramble shRNA lentiviruses were named 231 shNC. BC cell lines MCF-7 and T47D stably overexpressing ERCC6L protein were constructed by infection with pHBLV-ERCC6L lentivirus (named MCF7 ERCC6L and T47D ERCC6L, respectively). Control cells were designated MCF7 NC and T47D NC. The effects of ERCC6L knockdown and overexpression were determined by Western blotting (Supplementary Fig. S2a). Then, proliferation assays, colony-forming assays, 3D-Matrigel culture models and Transwell assays were conducted to evaluate the functional role of ERCC6L in BC in vitro. Indeed, ERCC6L overexpression significantly promoted the proliferation (Fig. 2A), colony formation (Fig. 2B and Supplementary Fig. S2b) and cell growth in 3D Matrigel (Fig. 2C and Supplementary Fig. S2c) of both MCF-7 and T47D cells, whereas ERCC6L knockdown had an inhibitory effect on MDA-MB-231 cells. The EdU assay revealed that ERCC6L could enhance DNA synthesis. Conversely, ERCC6L knockdown in MDA-MB-231 cells resulted in the opposite effect (Fig. 2D-E). In addition, migration and invasion were suppressed in MDA-MB-231 cells with ERCC6L knockdown, whereas both were enhanced in MCF-7 and T47D cells with ERCC6L overexpression (Fig. 2F and Supplementary Fig. S2d). To further confirm the effects of ERCC6L in vivo, human BC xenografts in nude mice were established using 231 shERCC6L cells. Compared with that in the nude mice inoculated with control cells, tumor growth in nude mice inoculated with ERCC6L-knockdown 231 cells was significantly inhibited (Fig. 2G-H). The ERCC6L knockout efficiency of inoculated 231 cells is shown in Supplementary Fig. S2e. Altogether, ERCC6L is essential for tumor progression and can promote the development of breast cancer.

### ERCC6L accelerates the G_2_/M phase transition of BC cells by regulating the p53/p21/CDK1/Cyclin B and Aurora A/PLK/CDC25C/CDK1/Cyclin B pathways

**Fig. 3:**
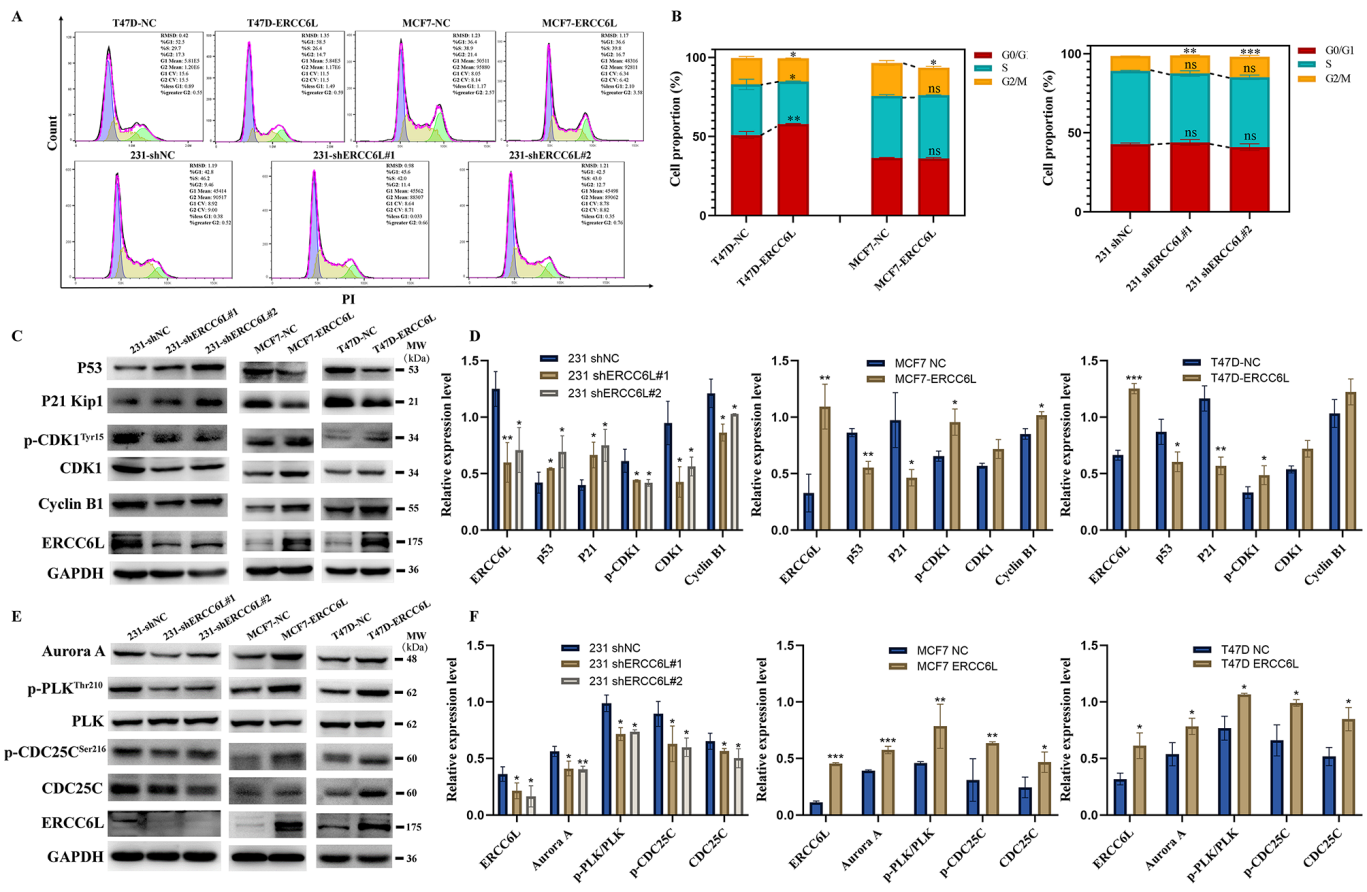
ERCC6L accelerates the G_2_/M phase transition of BC cells by regulating the p53/p21/CDK1/Cyclin B and Aurora A/PLK/CDC25C/CDK1/Cyclin B pathways. (**A**) ERCC6L overexpression reduced the proportion of G_2_/M phase cells in T47D and MCF-7 cells, while ERCC6L knockdown induced G_2_/M phase arrest. (**B**) Quantification of the flow cytometry cell cycle assay (n = 3). (**C**) The p53/p21/CDK1/Cyclin B signalling pathway in the G2/M checkpoint was regulated by ERCC6L. (**D**) Quantification of p53/p21/CDK1/Cyclin B signal pathway-related proteins. (**E**) The Aurora A/PLK/CDC25C/CDK1/Cyclin B signalling pathway in the G_2_/M checkpoint was regulated by ERCC6L. (**F**) Quantification of Aurora A/PLK/CDC25C/CDK1/Cyclin B signal pathway-related proteins. The results from Western blotting were representative of three independent experiments and then quantified by densitometry using Image J V1.8.0. Data are shown as the means ± SDs. Student’s t test or one-way ANOVA was applied to analyze the statistical significance. ***P < 0.001, **P < 0.01, *P < 0.05

ERCC6L is known as a protein involved in the mitotic process. To explore its regulatory role in the cell cycle, flow cytometry was carried out. Figure 3 A and B show that ERCC6L overexpression reduced the proportion of MCF-7 and T47D cells in the G_2_/M phase, which indicated an accelerated rate of cell mitosis. In contrast, ERCC6L knockdown induced significant G_2_/M phase arrest, which may further lead to the death of 231 cells. Accordingly, the RNA-Seq data in 231 shERCC6L cells also showed an enrichment in the apoptosis signal pathway (Supplementary Fig. S3a). DNA staining analysis showed that the nuclear morphogenesis of 231 shERCC6L cells changed significantly (nucleus condensation and fragmentation, the appearance of apoptotic bodies in the cytoplasm, etc.) (Supplementary Fig. S3b). Flow cytometry (FCM) analysis showed that knocking out ERCC6L increased the proportion of early and late apoptotic cells (Supplementary Fig. S3c). Based on the above results, ERCC6L may act as an important regulator in the cell cycle progression of BC cells. To identify the potential mechanisms, we checked proteins at the G_2_/M checkpoint using Western blotting. Figure 3 C and D showed that overexpression of ERCC6L increased the expression levels of CDK1 and Cyclin B and promoted the phosphorylation of CDK1 at Tyr 15, whereas ERCC6L knockdown led to adverse results. The Cyclin B-CDC2 (CDK1) complex, which is essential for G_2_ phase transition, is regulated by two parallel upstream cascades, Aurora A/PLK/CDC25C and p53/p21. Aurora A, an important member of the Aurora kinase family, is mainly responsible for regulating cell division by controlling centrosome duplication, segregation and maturation and spindle assembly. Aurora A has been demonstrated to be overexpressed in a variety of cancers, including breast cancer, and is closely related to malignant tumor progression [18]. Overexpression of ERCC6L downregulated the expression levels of p53 and p21, upregulated the levels of CDC25C and Aurora A, and promoted the phosphorylation of PLK at Thr 210 and CDC25C at Ser 216. Thus, ERCC6L regulated the cell cycle though the p53/p21/CDK1/Cyclin B and Aurora A/PLK/CDC25C/CDK1/Cyclin B pathways (Fig. 3E and F). Overall, these results suggested that ERCC6L accelerates the G_2_/M phase transition of BC cells by regulating the p53/p21/CDK1/Cyclin B and Aurora A/PLK/CDC25C/CDK1/Cyclin B pathways.

## ERCC6L is vital for proper mouse ductal network development

**Fig. 4:**
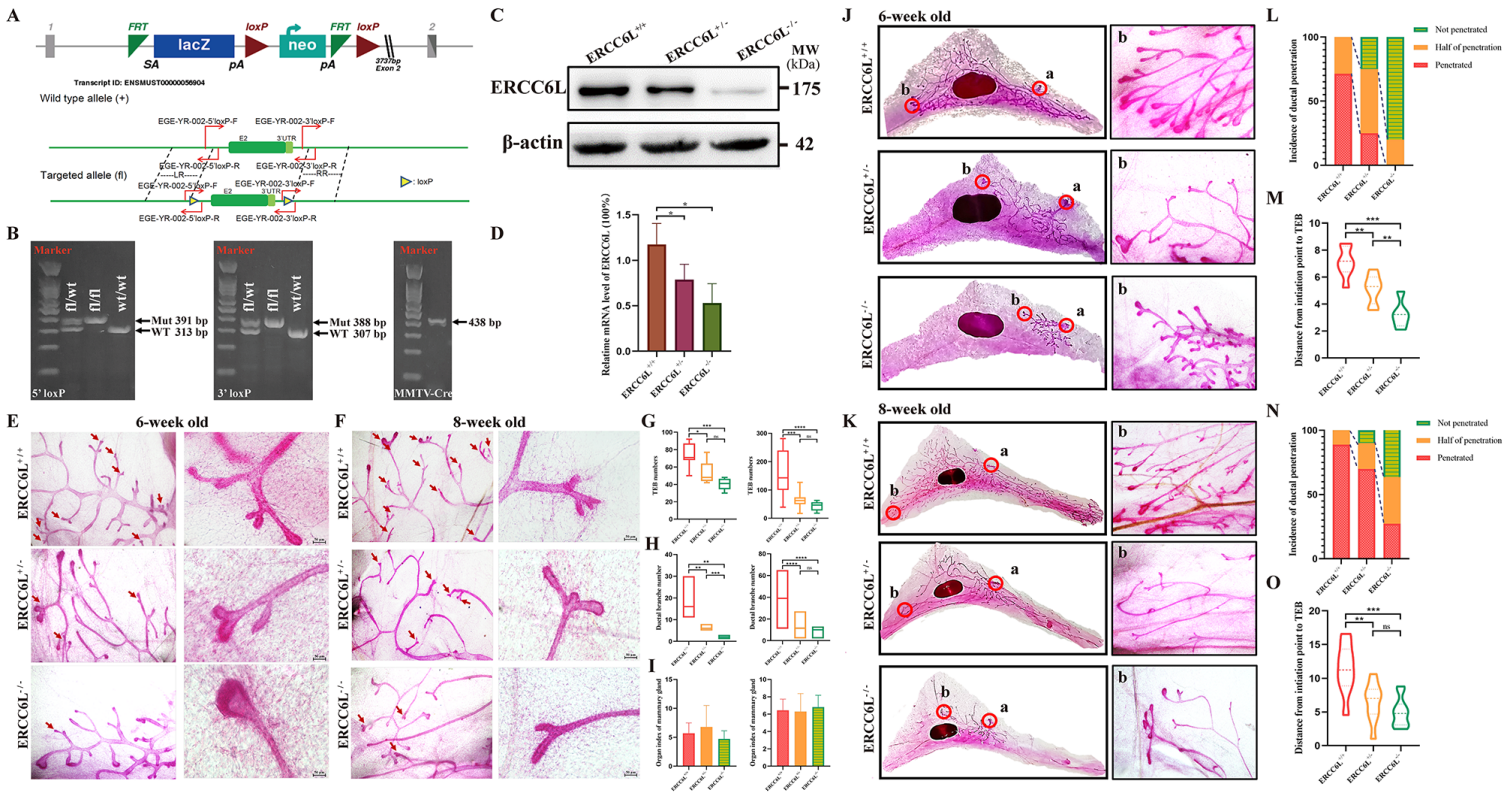
ERCC6L is vital for proper mouse ductal network development. (**A**) Schematic diagram of ERCC6L conditional knockout mouse construction. (**B**) Deletions of ERCC6L were confirmed by detecting genomic DNA extracted from the mouse tail using specific primers. (**C**) Extracted protein from the fourth inguinal mammary gland of mice was used for Western blotting, and the results confirmed ERCC6L deficiency. (**D**) The mRNA level of ERCC6L in the mammary gland was decreased in ERCC6L conditional knockout mice. (**E**-**F**) Representative images of mammary gland ductal TEBs and branches from 6- and 8-week-old ERCC6L^+/+^, ERCC6L^+/−^ and ERCC6L^−/−^ mice, respectively. Arrows represented mammary gland ductal TEBs and branches. (**G**) TEB numbers of 6- and 8-week-old mice. (**H**) Mammary gland ductal branches of 6- and 8-week-old mice. (**I**) Organ index of the mammary gland of 6- and 8-week-old mice. (**J**-**K**) Whole mount assay for the fourth inguinal mammary gland from 6- and 8-week-old ERCC6L^+/+^, ERCC6L^+/−^ and ERCC6L^−/−^ mice. “a” represented the initiation point and “b” represented the terminal TEBs. (**L**, **N**) Incidence of ductal penetration in 6- and 8-week-old mice. (**M**, **O**) Ductal penetration from the initiation point to the TEB of 6- and 8-week-old mice. For 6-week-old mice, the numbers of mammary glands used were ERCC6L^+/+^ (n = 7), ERCC6L^+/−^ (n = 8) and ERCC6L^−/−^ (n = 6). For 8-week-old mice, the numbers of mammary glands used were ERCC6L^+/+^ (n = 9), ERCC6L^+/−^ (n = 10) and ERCC6L^−/−^ (n = 9). Data are represented as the mean ± SD. Student’s t test and two-way ANOVA were applied to analyze the data. A *P* value < 0.05 was considered statistically significant. ***P < 0.001, **P < 0.01, *P < 0.05

To assess the function of ERCC6L in mammary gland development, a specific conditional knockout model of ERCC6L in the mammary gland was generated through Cre/loxP-mediated recombination (Fig. 4A). Floxed ERCC6L mice were intercrossed with MMTV-Cre mice, and then the ERCC6L gene was specifically deleted due to the Cre recombinase expressed in the hybrid mice (Supplementary Fig. S4a). Specific primers for various ERCC6L alleles (i.e., wild-type, floxed and deleted) and for Cre were used in the genotyping of female offspring. They were designated ERCC6L^fl/fl^ Cre^+/−^ (ERCC6L^−/−^), ERCC6L^fl/wt^ Cre^+/−^ (ERCC6L^+/−^) and ERCC6L^wt/wt^ Cre^+/−^ (ERCC6L^+/+^) (Fig. 4B). The deletion efficiency of ERCC6L in the mammary glands was confirmed by quantitative RT‒qPCR, Western blotting and IHC of mammary gland tissues (Fig. 4C-D and Supplementary Fig. S4b).

Then, we dissected the fourth inguinal mammary glands from mice of three genotypes aged 6, 8, and 12 weeks, which were subsequently weighed and stained with alum carmine. Mouse mammary gland terminal end buds (TEB), having a high ability of division and proliferation, are composed of cap cells, myoepithelial cells and luminal epithelial cells and are closely related to mammogenesis. When the mammary gland enters a period of rapid development, the duct can extend towards the mammary fat pad under the guidance of the TEB and then form secondary and tertiary branches [19]. Therefore, TEB number and branching complexity can indicate the developmental status of the mammary gland to a certain extent. As shown in Fig. 4E-H, the TEB number and the branching number of 6- and 8-week-old ERCC6L^+/+^ mice were both higher than those of ERCC6L^+/−^ and ERCC6L^−/−^ mice aged 6 and 8 weeks. Furthermore, the numbers of TEBs and branches in 6- and 8-week-old ERCC6L^+/−^ mice were also significantly greater than those in ERCC6L^−/−^ mice. However, there was no difference in the mammary gland organ index among the three genotypes of mice (Fig. 4I), which ruled out the impact of mouse self-growth on mammary duct development.

Additionally, the length of the straight line from the initiation point to the TEB represents ductal outgrowth to some extent. Figure 4 J-K showed that the TEBs of most ERCC6L^+/+^ mice aged 6 and 8 weeks penetrated the lymph node (LN). However, at 6 weeks, the TEBs of most ERCC6L^+/−^ mice were located near the LN, while those of most ERCC6L^−/−^ mice remained at the initiation site of the ductal and even approached the LN at 8 weeks. Moreover, regardless of whether at 6 or 8 weeks, mammary fat pads of ERCC6L^+/+^ mice had longer ductal outgrowth than ERCC6L^+/−^ and ERCC6L^−/−^ mice (Fig. 4L-O). Nonetheless, there were no significant differences in mammary gland organ index, TEB number, TEB bifurcation number and duct distance among the different genotypes aged 12 weeks (Supplementary Fig. S4c). It is speculated that the effect of their self-growth on mammoplasia exceeds the regulatory effect of ERCC6L knockout on mammogenesis in 12-week-old mice. Collectively, these results indicated that the deletion of ERCC6L could cause dysplasia of the mammary gland.

Apart from the organ index of the mammary gland, other organ indices were also detected in three genotypes of mice aged 6 and 8 weeks, including the heart, liver, spleen, lung, and kidney. However, there were no significant discrepancies in different organ indices between these genotype mice (Supplementary Fig. S4d), except for the spleen organ index in 8-week-old mice (Supplementary Fig. S4e-f). Considering that the spleen is a vital immune organ, we speculated that ERCC6L had a certain correlation with immunity. Thus, bioinformatics analyses and immunofluorescence (IF) staining of the spleen in mice of different genotypes at the age of 8 weeks were carried out. ERCC6L was closely related to B cells and CD8^+^ T cells, especially macrophages. The deletion of ERCC6L induced an increase in the number of CD8^+^ T cells and macrophages (Supplementary Fig. S4g-h), which was also confirmed by IF (Supplementary Fig. S4i). In summary, ERCC6L may affect the immunity of mice. However, whether the retention of immune cells in the spleen of ERCC6L^−/−^ mice exerted positive or negative immune regulation is still worth further investigation.

## Effect of ERCC6L deletion on MMTV/PyMT tumor onset and growth

**Fig. 5:**
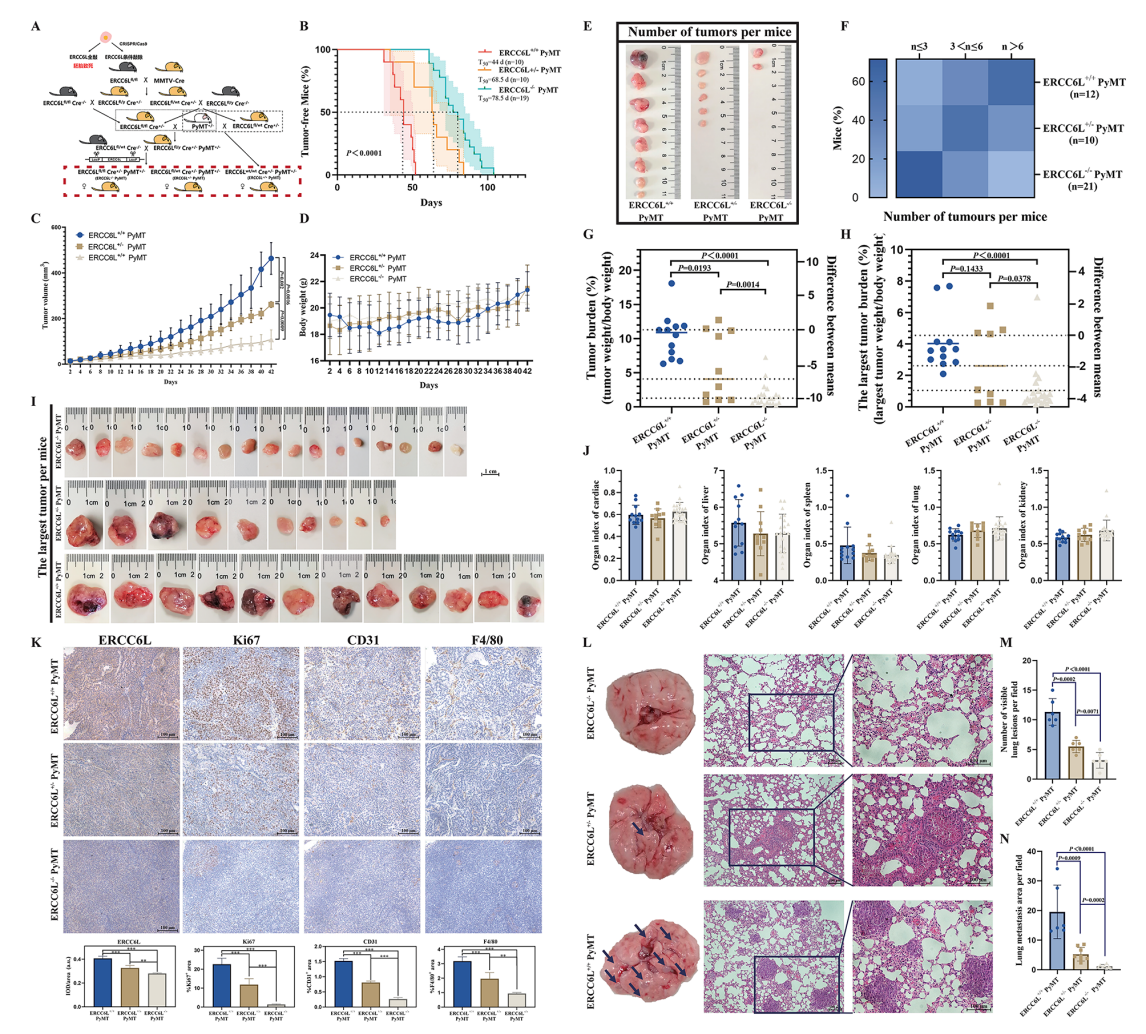
Effect of ERCC6L deletion on MMTV/PyMT tumor onset and growth. (**A**) Schematic illustrating the genetic hybridization process used for generating PyMT mice with different genotypes. (**B**) The tumor occurrence of mice was monitored by palpation on a weekly basis and was represented on a Kaplan‒Meier survival curve. T_50_ represented the median time for each group of mice to develop palpable tumors. The number of mice in each group was ERCC6L^+/+^ PyMT (n = 10), ERCC6L^+/−^ PyMT (n = 10) and ERCC6L^−/−^ PyMT (n = 19). (**C**) The tumor volume of each group of mice was recorded within 42 days from the onset of promotable tumors. (**D**) Body weight of mice in each group over 42 days. (**E**) Representative image of the tumor burden in each group of mice. Tumor multiplicity (**F**), tumor burden (**G**) and the largest tumor burden (**H**) for each group of mice were measured after euthanasia. The number of mice in each group was ERCC6L^+/+^ PyMT (n = 12), ERCC6L^+/−^ PyMT (n = 10) and ERCC6L^−/−^ PyMT (n = 21). (**I**) The largest tumor per mouse in each group. The scale bar is 1 cm. (**J**) Organ index of body weight, heart, liver, spleen, lung, and kidney. (**K**) IHC for ERCC6L, proliferation marker Ki67, immune infiltration markers F4/80, and angiogenesis marker CD31. (**L**) Representative images of lung metastasis in different groups of mice. Number (**M**) and area (**N**) of pulmonary metastatic nodules. Means ± SDs were represented, and Student’s t test and one-way ANOVA were applied to compare the data

To assess the role of ERCC6L in breast tumorigenesis in vivo, a spontaneous breast cancer mouse model was constructed by crossing mice of three genotypes with MMTV-PyMT mice, named ERCC6L^+/+^ PyMT, ERCC6L^+/−^ PyMT and ERCC6L^−/−^ PyMT (Fig. 5A). MMTV-PyMT mice are a widely applied model for BC studies due to their ability to generate spontaneous breast cancer tumors that are closely related to the progression and morphology of human breast cancer [20]. PyMT could be identified by specific primers in this study (Supplementary Fig. S5a). Then, we used palpation to investigate mammary tumor onset in female mice and observed that both ERCC6L^−/−^ PyMT and ERCC6L^+/−^ PyMT mice had slowed mammary tumor onset, with median tumor latencies (T_50_) of 78.5 days (n = 19) and 68.5 days (n = 10), respectively, which were significantly higher than those of ERCC6L^+/+^ PyMT mice (T_50_ = 44 days; n = 10) (Fig. 5B). Meanwhile, whole mount staining of the mammary glands of 8-week-old nontumor-bearing mice showed that ERCC6L deletion could inhibit early mammary hyperplastic lesions to some extent (Supplementary Fig. S5b).

The tumor growth rate of different genotypes of tumor-bearing mice was monitored to determine the effect of ERCC6L on tumor development. The results showed that the tumor growth speed in ERCC6L^+/+^ PyMT mice was significantly faster than that in mice with the ERCC6L deletion, and there was also a statistically significant difference in the tumor growth rate between ERCC6L^+/−^ PyMT (*P* = 0.002) and ERCC6L^−/−^ PyMT mice (*P* = 0.0016) without a significant change in body weight (Fig. 5C-D).

At 16 weeks, the mice were dissected, and the tumor multiplicity (the number of tumors per mouse), tumor burden, and individual tumor weight (the largest tumor weight) per mouse were counted. Compared with that in the ERCC6L^+/+^ PyMT group, the number of tumors was significantly reduced in the ERCC6L deletion groups, especially in the ERCC6L^−/−^ PyMT group (Fig. 5E). As shown in Fig. 5F and Supplementary Fig. S5c, there were fewer than three tumors in most ERCC6L^−/−^ PyMT mice, while ERCC6L^+/+^ PyMT mice mostly carried more than six tumors. In addition, ERCC6L deletion mice had a lower tumor burden and individual tumor weight than ERCC6L^+/+^ PyMT mice, with significant statistical discrepancies (Fig. 5G-I). However, there were no significant differences in the organ indices of the heart, liver, spleen, lung, and kidney (Fig. 5J). All these results suggested that ERCC6L deletion influences the occurrence and development of mammary gland tumors in vivo.

Subsequently, IHC was carried out in mammary gland tumor tissue samples from mice with diverse genotypes. The Immunolabelling markers Ki67, F4/80, and CD31, were used to evaluate cell proliferation, immune infiltration, and tumor angiogenesis in the three groups of mice. IHC staining clearly showed that the percentages of Ki67-positive, F4/80-positive and CD31-positive cells were significantly reduced in the ERCC6L deletion group compared with those in the ERCC6L^+/+^ PyMT group (Fig. 5K). We also measured the expression level of kinesin family member 4 (KIF4A) in tumor tissues, which also plays a role in chromosome disjunction during mitosis, and found that KIF4A was highly expressed in tumor tissues with high ERCC6L expression levels (Supplementary Fig. S5d). Pearson correlation analysis demonstrated that there was a strong positive correlation between ERCC6L and KIF4A (Supplementary Fig. S5e). These findings suggested that ERCC6L could facilitate malignant progression in mammary gland tumor growth.

Since the spontaneous tumors of MMTV/PyMT mice tend to metastasize to the lung at a later stage [21], our earlier data also showed that ERCC6L could promote the migration and invasion of BC cells. Therefore, to investigate whether ERCC6L deletion played a causal role in metastasis suppression in vivo, the lungs of each mouse were examined during dissection. Notably, not every mammary gland tumor-bearing mouse experienced lung metastasis, but among mice with lung metastasis of different genotypes, there were significant differences in the number and size of metastases (Fig. 5L-N). In summary, ERCC6L deletion inhibited the onset and growth of MMTV/PyMT tumors.

## KIF4A is closely related to ERCC6L in BC

**Fig. 6:**
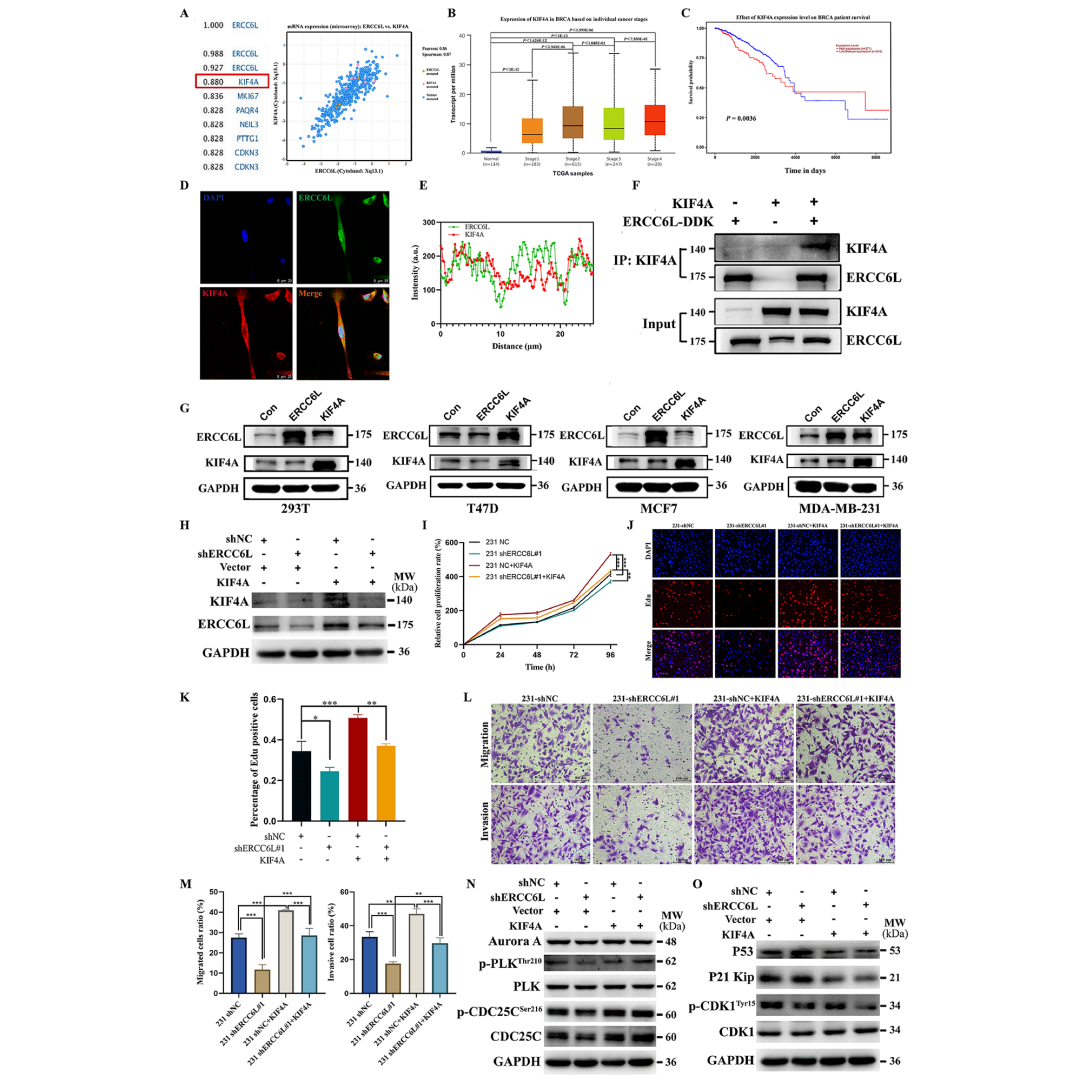
KIF4A is closely related to ERCC6L in the malignant progression of BC. (**A**) An *in silico* assay of TCGA data showed that KIF4A was closely related to ERCC6L. (**B**) Expression of KIF4A in BC based on individual cancer stages and (**C**) effect of KIF4A expression level on BC patient survival in the UALCAN database using TCGA samples. (**D**) Confocal immunofluorescence images of ERCC6L and KIF4A. (**E**) Fluorescence colocalization analysis of KIF4A and ERCC6L using Image J V1.8.0. (**F**) Co-IP assay showed that ERCC6L can interact with KIF4A. (**G**) Transfection of ERCC6L and KIF4A plasmids into 293T, T47D, MCF-7 and MDA-MB-453 cells was performed to detect changes in the expression levels of the two proteins. (**H**) KIF4A can upregulate ERCC6L expression levels. KIF4A reversed the inhibitory effects of ERCC6L deletion on cell growth (**I**), DNA synthesis (**J**-**K**), migration and invasion (**L**-**M**) in 231 cells. The scale bar of images is 100 μm. All experiments were performed in triplicate (n = 3). (**N**-**O**) MDA-MB-231 cells were transfected with vector or KIF4A plasmid for 48 h, followed by Western blotting with corresponding antibodies. Means ± SDs were represented, and one-way ANOVA was applied to compare the data. ***P < 0.001, **P < 0.01, *P < 0.05

As mentioned earlier, a strong correlation was observed between the expression of KIF4A and ERCC6L in mammary gland tumor tissues. Thus, we focused on KIF4A in BC. KIF4A is a member of the kinesin superfamily proteins (KIFs), whose members are involved in the transport of membranous organelles, protein complexes, mRNA and other substances [22]. Furthermore, KIF4A is also an ATP-dependent microtubule-based motor protein that participates in chromosome arm condensation and the maintenance of chromosome integrity during mitosis. In addition, it has been reported that KIF4A was involved in central spindle formation and centrosome assembly prior to cytokinesis [23, 24]. In summary, KIF4A is crucial for maintaining proper mitosis, and its abnormal expression has been reported in various tumors [25–28]. To verify the potential association of KIF4A with ERCC6L in BC, an *in silico* assay was carried out and showed that the expression of KIF4A was strongly related to ERCC6L (Pearson value = 0.880) (Fig. 6A). Moreover, KIF4A was observed to have a similar expression trend to ERCC6L in different types of BC (Supplementary Fig. S6a) and was highly expressed in high-grade tumors (Fig. 6B). This high expression of KIF4A was associated with poor outcomes of early-stage BC patients (Fig. 6C). To explore the possible effect of KIF4A on BC cells, three siRNAs were purchased, and two siRNAs with high silencing efficiency were selected for experiments (Supplementary Fig. S6b). Interference with the expression of KIF4A by siRNAs inhibited the growth and migration of MDA-MB-231 cells (Supplementary Fig. S6c-d). These results indicated that KIF4A was also involved in the progression of BC.

Nonetheless, the role of ERCC6L and KIF4A in tumor progression remains unclear. To determine whether there is an interaction between KIF4A and ERCC6L, immunofluorescence assays and Co-IP were carried out. First, the confocal immunofluorescence assay confirmed the colocalization of KIF4A and ERCC6L (Fig. 6D-E and Supplementary Fig. S6e). Then, ERCC6L-DDK and KIF4A plasmids were transfected into 293T cells separately or jointly for Co-IP detection. As shown in Fig. 6F, if only the ERCC6L-DDK plasmid was transfected, anti-DDK magnetic beads could pull down the overexpressed ERCC6L-DDK protein, which was detected in subsequent Western blotting. However, due to the low background expression of KIF4A, very little KIF4A could be detected. After simultaneous transfection of both plasmids, high KIF4A content was observed, indicating direct binding between KIF4A and ERCC6L. Subsequently, a Western blotting assay was performed in protein extracts from various cells that received transfection with KIF4A and ERCC6L plasmids and showed that compared with the control group, KIF4A overexpression significantly upregulated the protein level of ERCC6L, while there was no significant change in the protein level of KIF4A after the overexpression of ERCC6L (Fig. 6G). Similarly, as shown in Supplementary Fig. S6f, KIF4A levels were also measured in MCF-7 and T47D cells with stable overexpression of ERCC6L and MDA-MB-231 cells with stable knockdown of ERCC6L, and the results showed that overexpression or knockdown of ERCC6L had little effect on KIF4A levels. However, after transfection of the KIF4A plasmid in T47D, MCF7 and 231 cells, ERCC6L protein and mRNA levels were upregulated (Supplementary Fig. S6g-h), which provided further elucidation of the potential relationship between ERCC6L and KIF4A. Therefore, we hypothesized that there was an interaction between KIF4A and ERCC6L.

**Fig. 7:**
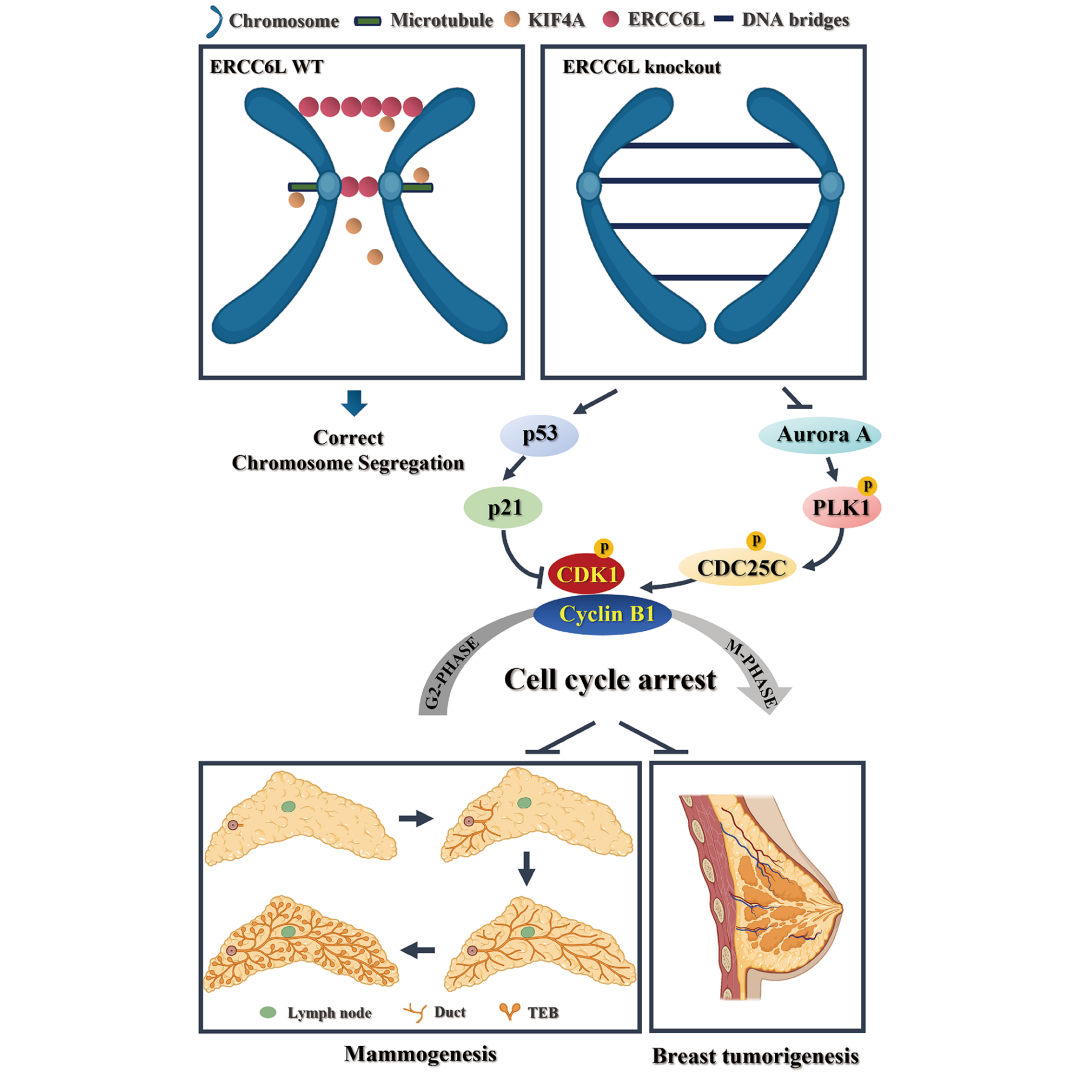
ERCC6L knockout inhibited mammogenesis and the tumorigenesis and development of breast cancer. In normal cells, ERCC6L, a DNA transposase, can decompose ultrafine anaphase DNA bridges to ensure correct chromosomal segregation during mitosis. KIF4A, as a chromosomal kinesin, may have a synergistic effect with ERCC6L in mitosis. The deletion of ERCC6L could induce the accumulation of DNA bridges and lead to chromosomal instability, which promotes p53/p21 activation and inhibits the Aurora A/PLK1/CDC25C signalling pathway, thereby leading to cell cycle arrest, which hinders mammary gland development and breast tumorigenesis

To further validate the regulatory role of KIF4A on ERCC6L and whether KIF4A exerts its effect on cell functions and downstream pathways through ERCC6L, we transfected the KIF4A plasmid into 231 shERCC6L and 231 shNC cells. The expression levels of KIF4A and ERCC6L in 231 cells are shown in Fig. 6H. The proliferation, EdU and Transwell assays all showed that KIF4A overexpression could further promote the growth, proliferation, migration and invasion of 231 shNC cells and effectively reduce the inhibitory effect of ERCC6L knockout (Fig. 6I-M). Western blotting analysis also revealed that KIF4A overexpression could upregulate the expression of CDC25C and the phosphorylation of PLK, CDK1, and CDC25C, as well as downregulate the levels of p53 and p21 in both 231 shNC and 231 shERCC6L cells (Fig. 6N-O). Collectively, these results suggest that ERCC6L interacts with KIF4A, which facilitates the malignant progression of BC together. A representative mechanism diagram is shown in Fig. 7.

## Discussion

BC, which is highly heterogeneous and can be divided into numerous subtypes, has received much attention due to its high incidence in women. Drug reactivity and patient prognosis also vary according to BC subtypes. However, there is still no cure for advanced metastatic breast cancer and TNBC. Thus, discovering new potential targets is of great significance for the development of targeted drugs and prognosis improvement of patients. In this study, ERCC6L was found to be highly expressed in BC patients, and its expression level was positively correlated with the malignant grade of the tumor and the poor prognosis of patients, which to some extent suggested that ERCC6L played an essential role in the malignant progression of BC. Moreover, a higher expression level of ERCC6L was discovered in TNBC patient tissues and cell lines than in the tissues and cell lines of patients with other subtypes, indicating the potential value of ERCC6L in TNBC. Cell experiments in vitro were also performed and found that ERCC6L could facilitate the malignant progression of BC cells by promoting cell proliferation, migration and invasion. Knocking down ERCC6L significantly inhibited tumor growth in nude mice. In summary, using both in vitro cell culture and in vivo nude mouse models, we demonstrated the important role of ERCC6L in breast tumorigenesis. Taken together, our results indicated that ERCC6L was a novel, potential target that urgently needed to be explored.

A few studies have indicated that the knockdown of ERCC6L could induce cell cycle arrest, chromosome instability, and cell death in breast cancer cell lines. However, to the best of our knowledge, there is currently no in vivo study that directly examined the role of ERCC6L in breast tumorigenesis using ERCC6L conditional knockout mice. In our study, we explored the effects of ERCC6L on mammary gland development using an ERCC6L conditional knockout mouse model for the first time. Our results first showed that ERCC6L deletion significantly reduced the number of TEBs and ductal branches and significantly inhibited the extension of mammary branch ducts into the fat pad in both 6-week-old and 8-week-old mice, which suggested mammary dysplasia. However, mammary gland development in 12-week-old mice was not affected by ERCC6L deletion, and it is speculated that the effect on mouse self-growth and hormone levels exceeded the regulatory effect of ERCC6L on development. Interestingly, splenomegaly was found in 8-week-old ERCC6L^−/−^ mice, as indicated by increased infiltration of T cells, especially macrophages, in splenic tissues. It is possible that ERCC6L deletion mediated the generation of cells with a complex karyotype (aneuploid), which was reported to induce self-elimination by the immune system [29]. However, the potential relationship between ERCC6L and immunity has not been reported, so further confirmation and exploration are still needed.

Notably, ERCC6L, a key mitotic checkpoint-associated protein, is theorized to induce increased chromosome mis-segregation and chromosomal instability (CIN) when absent, which can contribute to tumorigenesis [30]. However, ERCC6L^−/−^ and ERCC6L^+/−^ mice did not develop mammary gland tumors, which suggested that ERCC6L knockout does not induce tumorigenesis alone and might not be a breast tumor susceptibility gene such as *BRCA1/2*. However, when crossed with MMTV-PyMT mice, ERCC6L knockout significantly suppressed mammary tumorigenesis. It is possible that, because PyMT itself could induce breast tumorigenesis, the loss of ERCC6L caused PyMT-mediated mammary gland tumor cells to undergo G_2_/M phase checkpoint disorder. Therefore, they could not repair the damaged DNA and directly enter mitosis, resulting in “mitotic catastrophe”, which caused aneuploidy, micronuclei, etc., and eventually led to tumor cell death. This was consistent with the results verified by Huang et al. [17] in breast cancer cell lines in vitro.

Lung metastasis has been observed in a high proportion of MMTV-PyMT mice [21]. Therefore, we also investigated the effect of ERCC6L on lung metastasis of mammary gland tumors. ERCC6L knockdown was first found to significantly suppress the number and area of lung metastases in tumor-bearing mice. However, further in-depth exploration is still needed to determine the role of ERCC6L in lung metastasis.

Another interesting finding in our study was that there was a direct interaction between ERCC6L and KIF4A. However, it remains unclear what consequences could be mediated by the interaction between ERCC6L and KIF4A. Moreover, ERCC6L and KIF4A may synergistically promote breast cancer cell proliferation, migration and invasion. However, how KIF4A regulates ERCC6L levels remains unknown. It is worth mentioning that Mazumdar et al. [31] found that knockdown of KIF4 resulted in changes in the mRNA levels of more than 500 genes and could increase the expression of normally silent repetitive elements in heterochromatin domains. This indicates that KIF4 could be involved in transcriptional regulation, and this mechanism of transcriptional control was related to the interactions between proteins such as poly ADP-ribose polymerase 1 (PARP-1), DNA methyltransferase 3B (DNMT3B) and the histone deacetylase HDAC1 [32]. Therefore, KIF4A may indirectly regulate the level of ERCC6L by regulating some related proteins. In summary, various studies have suggested that KIF4A was strongly associated with ERCC6L. However, the relationship between them still deserves further investigation.

Mechanistically, ERCC6L has been reported to act as a binding partner and substrate of polo-like kinases (PLKs), including PLK1, which is involved in centromeric chromatin remodelling [33, 34]. PLK1 is a key kinase for the DNA damage checkpoint [35]. Hence, we investigated the role of ERCC6L in cell cycle regulation and found that ERCC6L knockdown suppressed the activation of the PLK/CDC25C/CDK1/Cyclin B signalling pathway, thereby mediating cell cycle arrest. Furthermore, another important pathway for the DNA damage checkpoint, p53/p21/CDK1/Cyclin B, was significantly regulated in ERCC6L knockdown cells.

## Conclusion

In summary, we first demonstrated the role of ERCC6L in breast tumorigenesis and development in vivo using the ERCC6L conditional knockout mouse model, that is, ERCC6L is required for mammary gland tumorigenesis induced by MMTV-PyMT. In addition, ERCC6L, as a tumor promoter, could promote the proliferation, migration and invasion of breast cancer cells and accelerate cell cycle progression by activating the PLK/CDC25C/CDK1/Cyclin B and p53/p21/CDK1/Cyclin B signalling pathways. It is worth mentioning that ERCC6L was also demonstrated to have a close relationship with KIF4A, including interactions and regulatory relationships, which might be correlated with the mitotic process. Overall, targeting ERCC6L could be a promising strategy for the treatment of BC.

### Electronic supplementary material

Below is the link to the electronic supplementary material.

Supplementary Material 1

Supplementary Material 2

Supplementary Material 3

Supplementary Material 4

Supplementary Material 5

Supplementary Material 6

Supplementary Material 7

Supplementary Material 8

## Data Availability

The datasets supporting the conclusions of this article are available from the corresponding author upon reasonable request. All data in this study are included in this published article, including its supplementary information files. The datasets generated in this study are available in the TCGA (https://portal.gdc.cancer.gov/).

## Abbreviations

BC: Breast cancer
TNBC: Triple-negative breast cancer
PARP: Poly ADP-ribose polymerase
ERCC6L: ERCC Exception Repair 6 Like
PICH: PLK1-interacting checkpoint helicase
UFBs: Ultrafine DNA bridges
PyMT: Polyomavirus middle T antigen
KIF4A: Kinesin family member 4
TEB: Terminal end buds
LN: Lymph node
DNMT3B: DNA methyltransferase 3B
PLKs: Polo-like kinases
