# Supplementary material for: ERCC6L facilitates the onset of mammary neoplasia and promotes the high malignance of breast cancer by accelerating the cell cycle
Source: J Exp Clin Cancer Res. 2023 Sep 4;42:227. doi: 10.1186/s13046-023-02806-x (PMC10478442; doi:10.1186/s13046-023-02806-x)
Supplement: Supplementary file 1 — Supplementary Material 1 [file 13046_2023_2806_MOESM1_ESM.docx]

**Table 1**

**Primers used in the article**

| Name | Sequences |
| --- | --- |
| 5’loxP-F | TGAGCCTCAGCTGGTAAAGTGGTTG |
| 5’loxP-R | GCATGAAGGAGCCTCTGAAGCCAAT |
| 3’loxP-F | CACCTTCAGAAACACCAAGTGCAGC |
| 3’loxP-R | GTATGGACTTCCACCACCACACTGG |
| Cre-F | CATATTGGCAGAACGAAAACGC |
| Cre-R | CCTGTTTCACTATCCAGGTTACGG |
| PyMT-F | GGAAGCAAGTACTTCACAAGGG |
| PyMT-R | GGAAAGTCACTAGGAGCAGGG |
| ERCC6L-F | CCAAGGCTTAGCGGAAGTGGAGA |
| ERCC6L-R | AGCTCTCGGTAAAGCAGCAAGC |
| β-actin-F | AGGCCAACCGTGAAAAGATG |
| β-actin-R | TGGCGTGAGGGAGAGCATAG |
| KIF4A-F | TACTGCGGTGGAGCAAGAAG |
| KIF4A-R | CATCTGCGCTTGACGGAGAG |

**Antibodies**

| Name | Company | Reactivity | Source | Dilution ratio |
| --- | --- | --- | --- | --- |
| PICH | CST | H Mk | Rabbit | 1:1000/1:100 |
| ERCC6L | Proteintech | H M | Rabbit | 1:1000 |
| p53 | CST | H M R Hm Mk | Mouse | 1:1000 |
| p21 | CST | H Mk | Rabbit | 1:1000 |
| P-CDK1 (Tyr15) | CST | H M R Mk | Rabbit | 1:1000 |
| CDK1 | CST | H Mk | Mouse | 1:1000 |
| Cyclin B1 | CST | H M R Hm Mk | Rabbit | 1:1000 |
| Aurora A | CST | H | Rabbit | 1:1000 |
| P-PLK1 (Thr210) | CST | H | Rabbit | 1:1000 |
| PLK1 | CST | H R Mk | Rabbit | 1:1000 |
| P-CDC25C (Ser216) | CST | H Mk | Rabbit | 1:1000 |
| KIF4A | Abcam | H M | Rabbit | 1:1000 |
| β-actin | Proteintech | H M R P | Rabbit | 1:1000/1:100 |
| GAPDH | Proteintech | H M R | Mouse | 1:1000 |

H = Human; M = Mouse; R = Rat; Hm = Hamster; Mk = Monkey; P = Pig

**siRNA**

| Name | Sequences |
| --- | --- |
| KIF4A si#1 | GCAAGATCCTGAAAGAGAT |
| KIF4A si#2 | GCATTCTGTGAATGAGCAT |
| KIF4A si#3 | GGATGGTGATGGTGATGAT |
