# Supplementary figures and images for: ERCC6L facilitates the onset of mammary neoplasia and promotes the high malignance of breast cancer by accelerating the cell cycle
Source: J Exp Clin Cancer Res. 2023 Sep 4;42:227. doi: 10.1186/s13046-023-02806-x (PMC10478442; doi:10.1186/s13046-023-02806-x)

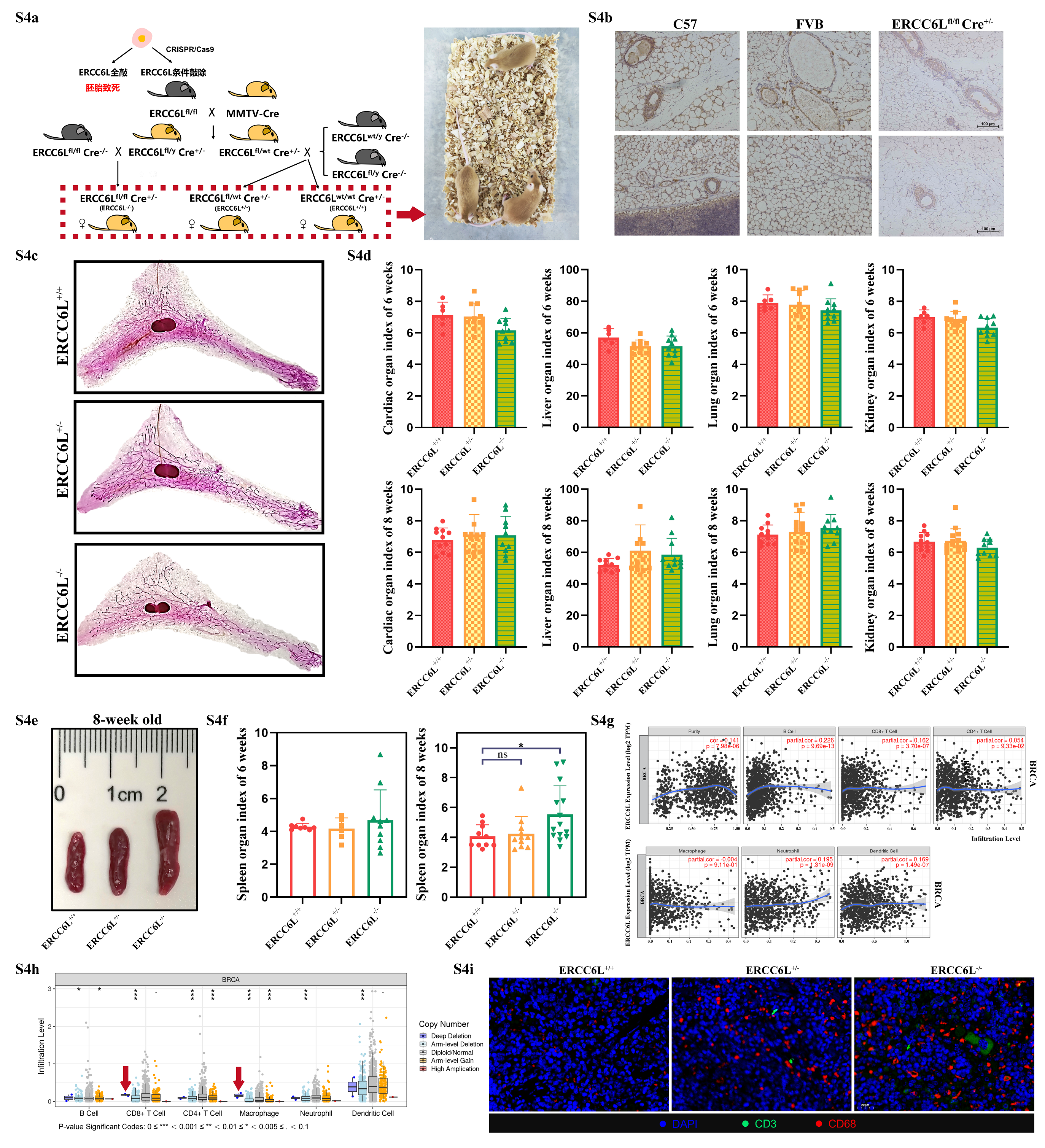

Supplement: Supplementary file 2 — Supplementary Material 2 [file 13046_2023_2806_MOESM2_ESM.png]

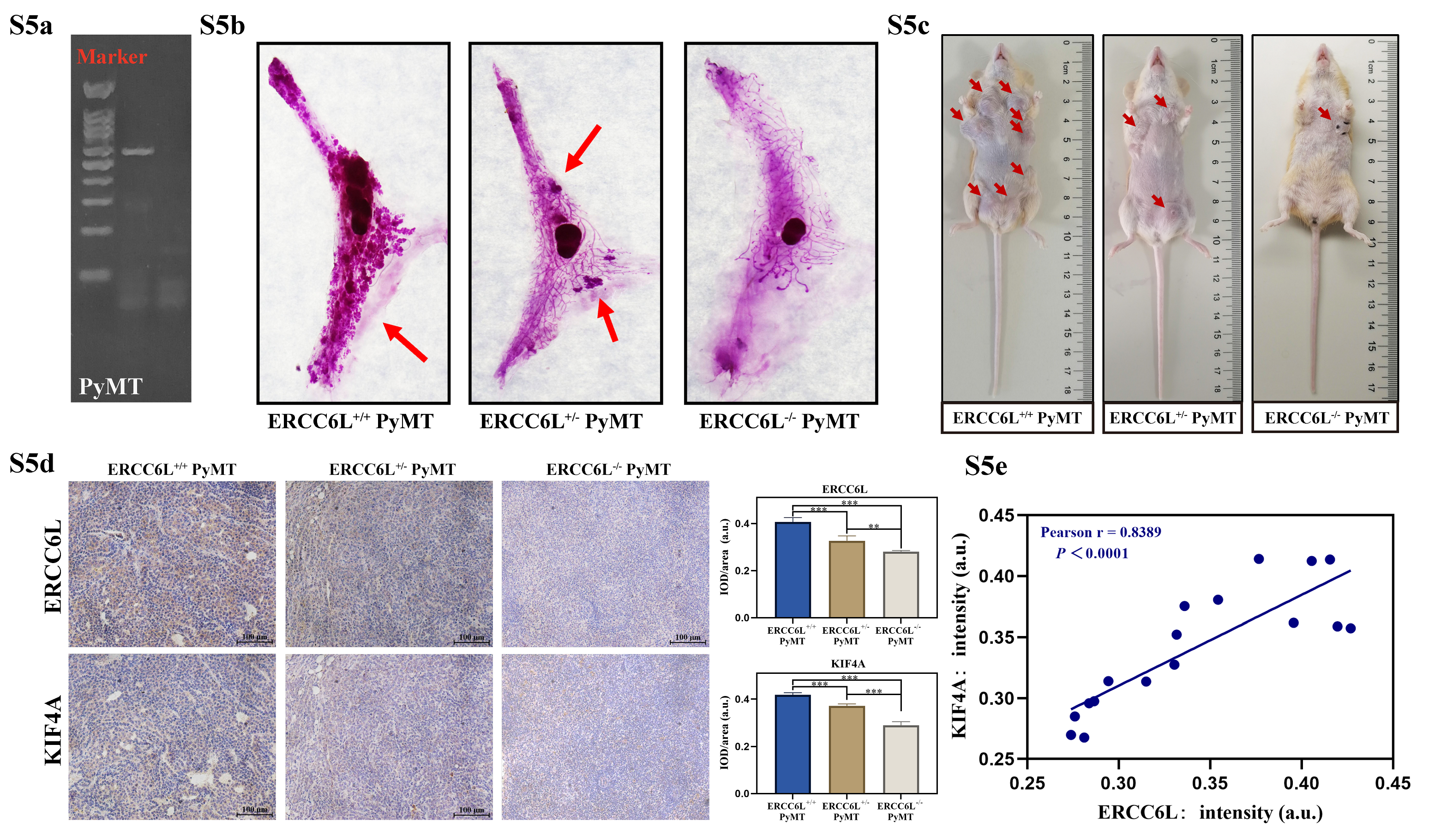

Supplement: Supplementary file 3 — Supplementary Material 3 [file 13046_2023_2806_MOESM3_ESM.png]

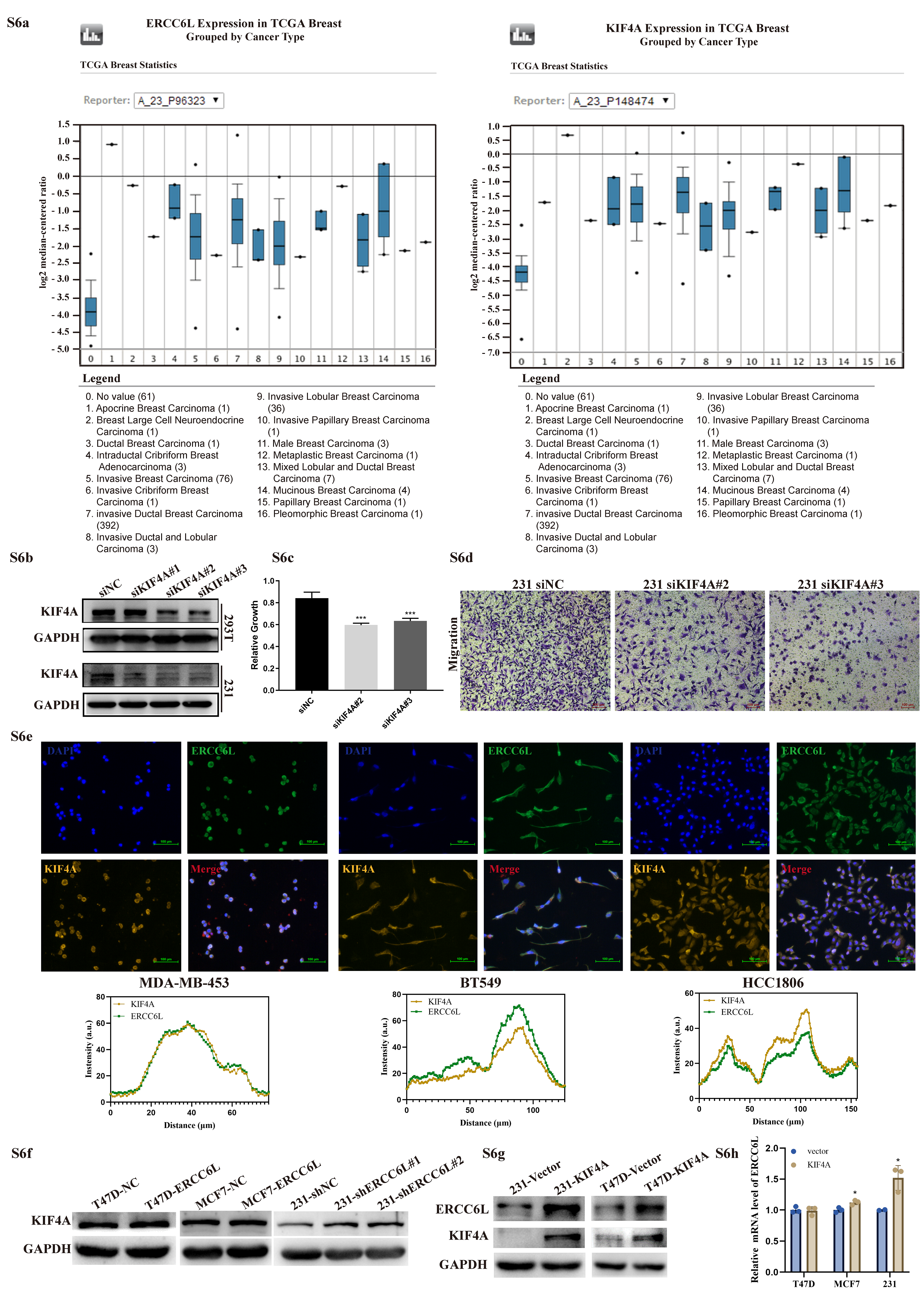

Supplement: Supplementary file 4 — Supplementary Material 4 [file 13046_2023_2806_MOESM4_ESM.png]

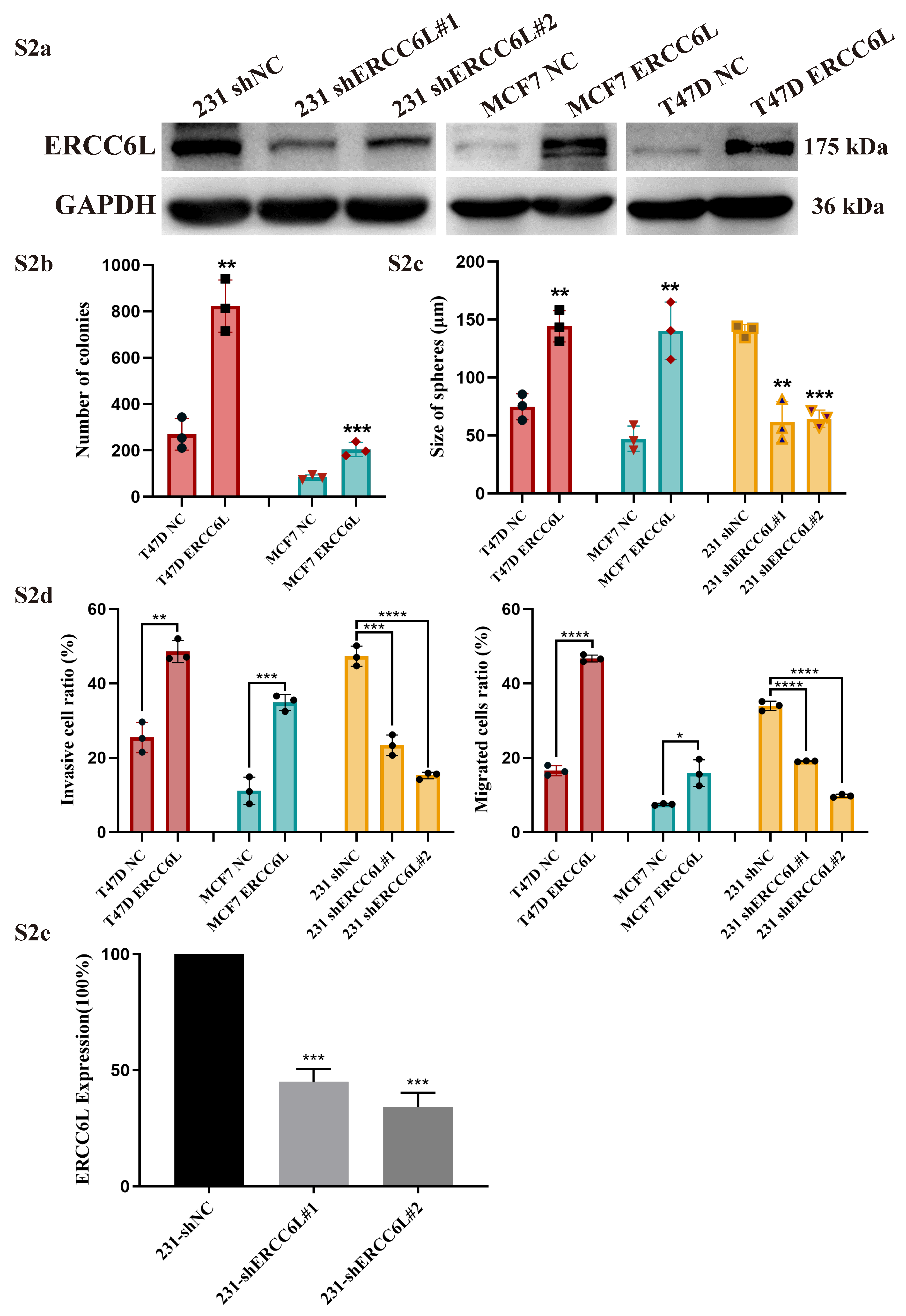

Supplement: Supplementary file 6 — Supplementary Material 6 [file 13046_2023_2806_MOESM6_ESM.png]

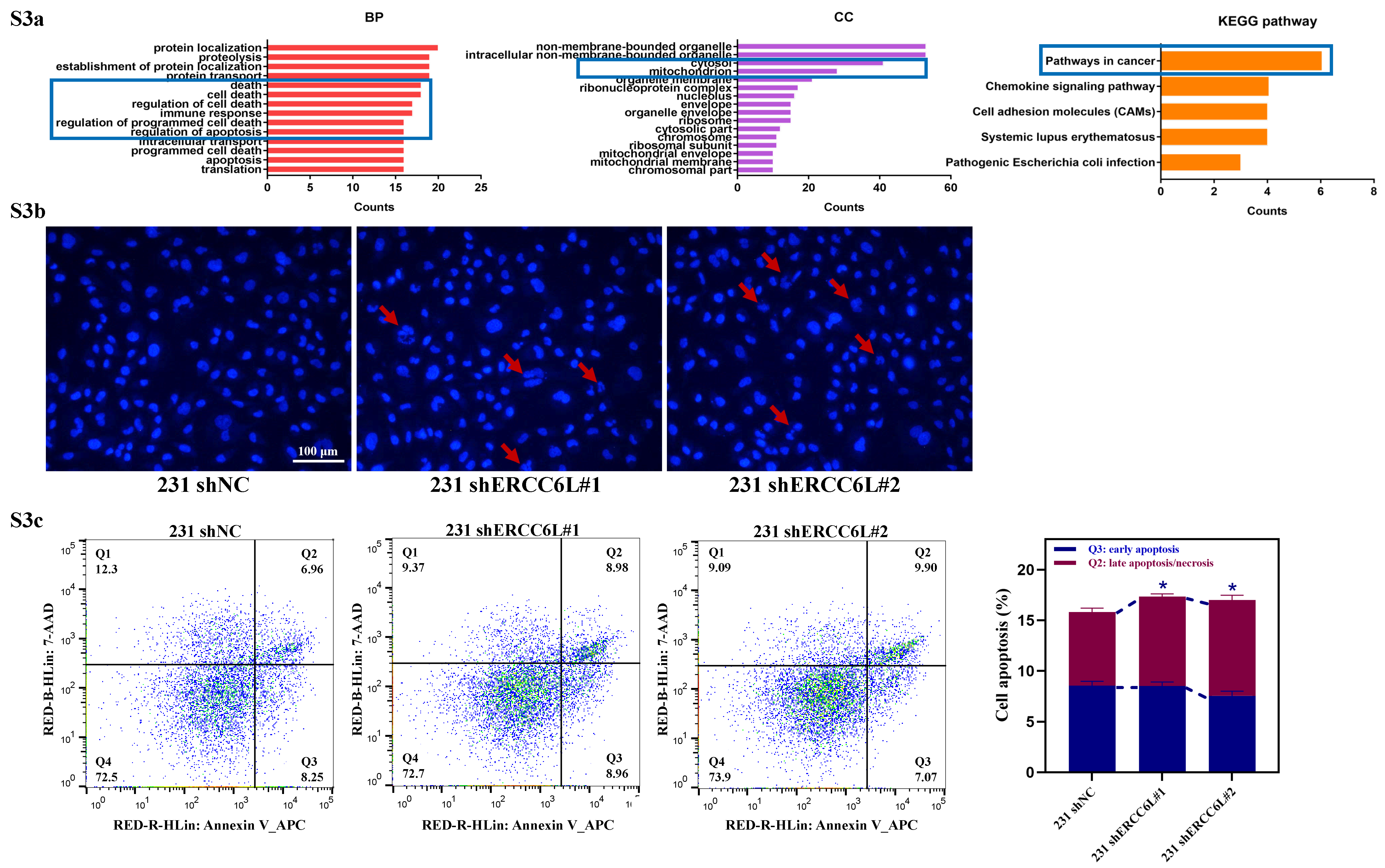

Supplement: Supplementary file 7 — Supplementary Material 7 [file 13046_2023_2806_MOESM7_ESM.png]
