# Supplementary material for: ERCC6L facilitates the onset of mammary neoplasia and promotes the high malignance of breast cancer by accelerating the cell cycle
Source: J Exp Clin Cancer Res. 2023 Sep 4;42:227. doi: 10.1186/s13046-023-02806-x (PMC10478442; doi:10.1186/s13046-023-02806-x)
Supplement: Supplementary file 8 — Supplementary Material 8 [file 13046_2023_2806_MOESM8_ESM.docx]

**Supplementary Figure 1.**

**S1a.** ERCC6L is highly expressed in BRCA compared to normal. **S1b-S1c.** Expression of ERCC6L in different subtypes of BRCA. **S1d-S1e.** The expression level of ERCC6L was positively correlated with the malignant grade of tumors. **S1f.** High expression of ERCC6L was associated with poor prognosis in BRCA patients. **S1g.** Quantitative analysis of ERCC6L expression level in the BC tissues (n=10) and adjacent breast tissues (n=10). The expression of ERCC6L was significantly higher in BC than in normal breast tissues (*P=*0.0027). **S1h.** Quantitative statistics on IRS score of ERCC6L in tissues from BC patients with different WHO grades. Student’s t-test or one-way ANOVA was applied to analyze the statistical significance.

**Supplementary Figure 2.**

**S2a.** ERCC6L was overexpressed in MCF-7 and T47D cells, while ERCC6L was knocked down in MDA-MB-231 cells. **S2b.** The quantification of colony formation of T47D and MCF7 cells (n=3). **S2c.** The quantification of the cell sphere size of T47D, MCF7 and MDA-MB-231 cells (n=3). **S2d.** Quantitative analysis of migrated and invasive cells (n=3). **S2e.** ERCC6L was significantly knocked down in MDA-MB-231 cells implanted in nude mice. Data were shown as Means ± SD. Student’s t-test or one-way ANOVA was applied to analyze the statistical significance. *****P* < 0.0001, ****P* < 0.001, ***P* < 0.01, **P* < 0.05.

**Supplementary Figure 3.**

**S3a.** RNA-Seq data showed that ERCC6L played a vital role in cell death. **S3b.** Effect of ERCC6L knockdown on the nuclei of MDA-MB-231 cells. The red arrow referred to apoptotic bodies. **S3c.** The deletion of ERCC6L could induce apoptosis of MDA-MB-231 cells by flow cytometry using Annexin V-APC/7-AAD double stain (n=3). Student’s t-test was applied to analyze the statistical significance. **P* < 0.05.

**Supplementary Figure 4.**

**S4a.** Genetic hybridization flowchart for obtaining ERCC6L knockout mice. **S4b.** IHC assay showed that ERCC6L was significantly deleted in ERCC6L^fl/fl^ Cre^+/-^ mice compared to the expression level of ERCC6L in parents. **S4c.** Whole mount assay showed that there was no significant difference in the development of mammary gland ducts among 12-week-old mice with diverse genotypes. **S4d.** Organ index of heart, liver, spleen, lung, kidney in 6- and 8-week-old mice. **S4e-S4f.** Splenomegaly was observed in ERCC6L^-/-^ mice at age of 8 weeks. **S4g.** Correlation analysis of ERCC6L expression and infiltration of various immune cells obtained from TIMER2.0 website (<http://timer.cistrome.org/>). **S4h.** ERCC6L deletion induced increased infiltration of CD8^+^ T cells and macrophages using TIMER2.0 website (<http://timer.cistrome.org/>). **S4i.** Immunofluorescence showed that the spleens of ERCC6L^-/-^ mice showed high levels of macrophage and T cells infiltration. Data were shown as Means ± SD. Student’s t test or one-way ANOVA was applied to analyze the statistical significance. **P* < 0.05.

**Supplementary Figure 5.**

**S5a.** PyMT can be identified by detecting genomic DNA using specific primers. **S5b.** Whole mount assay of the fourth inguinal mammary glands in 8-week-old PyMT mice. The arrows indicated precancerous hyperplasia. **S5c.** Representative images of tumor-bearing mice. ERCC6L^+/+^ PyMT mice had more tumors compared to ERCC6L conditional knockout mice. **S5d.** ERCC6L and KIF4A IHC staining of ERCC6L^+/+^ PyMT, ERCC6L^+/-^ PyMT and ERCC6L^-/-^ PyMT breast tumor sections. **S5e.** ERCC6L was strongly correlated with KIF4A. Data were shown as Means ± SD. Student’s t test or one-way ANOVA was applied to analyze the statistical significance. ****P* < 0.001, ***P* < 0.01, **P* < 0.05.

**Supplementary Figure 6.**

**S6a.** KIF4A had a similar expression profile with ERCC6L in BRCA using TCGA Breast statistics in Oncomine database. **S6b.** KIF4A could be significantly silenced using siKIF4A#2 and siKIF4A#3 in 293T and MDA-MB-231 cells (n=3). **S6d-S6c.** KIF4A knockdown could inhibit cell growth and migration in MDA-MB-231 cells. Data were shown as Means ± SD. Student’s t test or was applied to analyze the statistical significance. ****P* < 0.001. **S6e.** Immunofluorescence images of ERCC6L and KIF4A in MDA-MB-453, BT549 and HCC1806 cells. **S6f.** ERCC6L overexpression or knockdown had little effect on KIF4A level. **S6g-h.** ERCC6L protein and mRNA levels could be up-regulated in T47D, MCF-7 and MDA-MB-231 cells with the overexpression of KIF4A.
